# DNA Barcoding and Species Boundary Delimitation of Selected Species of Chinese Acridoidea (Orthoptera: Caelifera)

**DOI:** 10.1371/journal.pone.0082400

**Published:** 2013-12-20

**Authors:** Jianhua Huang, Aibing Zhang, Shaoli Mao, Yuan Huang

**Affiliations:** 1 College of Life Sciences, Shaanxi Normal University, Xi'an, People's Republic of China; 2 College of Life Sciences, Guangxi Normal University, Guilin, People's Republic of China; 3 College of Life Sciences, Capital Normal University, Beijing, People's Republic of China; Australian Museum, Australia

## Abstract

We tested the performance of DNA barcoding in Acridoidea and attempted to solve species boundary delimitation problems in selected groups using COI barcodes. Three analysis methods were applied to reconstruct the phylogeny. K2P distances were used to assess the overlap range between intraspecific variation and interspecific divergence. “Best match (BM)”, “best close match (BCM)”, “all species barcodes (ASB)” and “back-propagation neural networks (BP-based method)” were utilized to test the success rate of species identification. Phylogenetic species concept and network analysis were employed to delimitate the species boundary in eight selected species groups. The results demonstrated that the COI barcode region performed better in phylogenetic reconstruction at genus and species levels than at higher-levels, but showed a little improvement in resolving the higher-level relationships when the third base data or both first and third base data were excluded. Most overlaps and incorrect identifications may be due to imperfect taxonomy, indicating the critical role of taxonomic revision in DNA barcoding study. Species boundary delimitation confirmed the presence of oversplitting in six species groups and suggested that each group should be treated as a single species.

## Introduction

Acridoidea is the largest group in Orthoptera, with more than 1000 species known from China to date. However, imperfect taxonomy may exist in many species groups, especially in those with reduced tegmina. Many brachypterous and apterous new species have been described from China during the last thirty years based on minor differences which might in fact be normal intraspecific variations, and some of them are extremely close geographically to their nearest relatives, resulting in a possible oversplitting, i.e. morphotypes are inappropriately recognized as species [1]. Some efforts have been made to resolve these issues based on morphological study [2]–[4]. Of course, cryptic species may exist in some groups and additional evidence is needed to distinguish them from known close relatives.

Acrididae is the largest family in Acridoidea, including 24 subfamilies and some genera unassigned to any subfamily [5]. However, some family names, such as Catantopidae [6], Oedipodidae, Arcypteridae [7] and Gomphoceridae [8], have been defined from those subfamilies. Molecular evidence has long been used to resolve the phylogeny, evolutionary history and taxonomy of Acridoidea at different hierarchical levels. Besides some researches that discussed the monophyly of Caelifera as well as Acridoidea [9]–[21], there were also many investigations exploring the phylogenies and origins of their subordinate categories, including Acrididae [22]–[23], Pamphagidae [24]–[25], Catantopidae [26]–[28], Oedipodidae [29]–[30], Arcypteridae [31], Melanoplinae [32]–[35], Podisminae [36], Oedipodinae [37]–[39], Gomphocerinae [39]–[41], Proctolabinae [42], and so on. Because of the difference in taxon sampling, selection of genetic markers and analytical strategy, the results were frequently inconsistent to some extent among different case studies and the phylogenies remain unresolved in many taxa.

DNA barcoding is a diagnostic technique in which short DNA sequence(s) can be used for species identification [43]. It has attracted extensive attention from taxonomists all over the world. Using the standard 658-base fragment of the 5′ end of the mitochondrial gene cytochrome c oxidase subunit I (COI, cox1) as the barcode region for animals [44], the proponents of the approach suggest that at least 98% of congeneric species pairs of animals can be correctly identified through DNA barcoding [45]. Besides assigning specimens to known species, DNA barcoding also aids to highlight potential cryptic, synonymous and extinct species [46]–[49] as well as to match adults with immature specimens [50]. However, a lot of factors can affect the success rate of DNA barcoding, for example, the selection of gene fragment(s) [51], control of numts [52]–[53], monophyly of the known species [1], analytical methods [54]–[59], success rate of barcode amplification and sequencing [60], and so on. The effectiveness of barcoding is critically dependent upon species delineation [1]. Funk and Omland [61] found that about 23% of surveyed metazoan species were genetically polyphyletic or paraphyletic. Nearly all failures of species identification through DNA barcoding in cowries could be attributed to imperfect taxonomy (overlumping or overspliting) or incomplete lineage sorting [1], indicating that comprehensive taxonomic revision (species boundary delimitation) is crucial in the DNA barcoding studies.

Although there are a lot of successful examples in most insect orders using DNA barcoding to identify species [62]–[66], we have found only four studies concerning DNA barcoding of Acridoidea, one with positive result [67], one with a failed result [68], and two cases focused on the distribution patterns and coamplification of numts and their effects on DNA barcoding [52]–[53]. López *et al* [69] discussed the species boundary delimitation of threatened grasshopper species from the Canary Islands using both mitochondrial COI and nuclear ITS2 sequences.

To test the performance of DNA barcoding in Acridoidea on a larger scale, we examined patterns of CO1 divergence in 72 species/subspecies of grasshoppers mostly from China (*Schistocerca americana* and *Chorthippus parallelus* are not distributed in China and their sequences were downloaded from GenBank).

Taxon sampling was mainly focused on the following eight morphologically similar but taxonomically questionable species groups: (1) *Sinopodisma houshana+S. lushiensis+S. qinlingensis*; (2) *Pedopodisma tsinlingensis+P. funiushana+P. wudangshanensis*; (3) *Fruhstorferiola kulinga+F. huayinensis*; (4) *Shirakiacris shirakii+F. yunkweiensis*; (5) *Spathosternum prasiniferum prasiniferum*+*S. p. sinense*; (6) *Calliptamus italicus+C. abbreviatus*; (7) *Oedaleus decorus+O. asiaticus*; (8) *Oedaleus infernalis+O. manjius*.

For the first four groups, there is nearly no morphological difference found between species within each group, and the only criterion that can be used to identify species is geographical distribution information. Each group should be regarded as the same species according to the extremely high morphological similarity. *Shirakiacris yunkweiensis* was synonymized with *S. shirakii* [70], but this has not yet been accepted by Chinese acridologists, although there is no additional evidence to support their decision to retain the species status of *S. yunkweiensis* [6]. *Pedopodisma*, a genus endemic to China, was synonymized with *Sinopodisma* [71], but Storozhenko's treatment has not been accepted by Chinese acridologists [6] because tegmina are completely absent in *Pedopodisma* but distinct though reduced in *Sinopodisma*.

*Spathosternum sinense* was originally described as an independent species [72], then reduced to a race of *S. prasiniferum* [73], and even synonymized with the latter [74]. However, Grunshaw's synonymy was not accepted by other orthopterists, and *S. sinense* has been recognized as a subspecies of *S. prasiniferum* until now [5]. *S. prasiniferum prasiniferum* and *S. p. sinense* are usually distinguished from each other by their tegmen length, the former with tegmina reaching or slightly exceeding the apices of hind femora and the latter usually only reaching the middle of the hind femora. They may be allopatric and it remains unclear whether there is an overlap distributional zone. However, we did collect a few female individuals with long tegmina from the population of *S. p. sinense*, but never found males with this phenotype. It is not clear to which species/subspecies, *sinense* or *prasiniferum*, these rare individuals with long tegmina but from the population of *S. p. sinense* should be assigned. Whether these two taxa are species or subspecies requires clarification.

*Calliptamus italicus* is widely distributed in Europe, north Africa, central and west Asia as well as northwest China (Xinjiang and Qinghai Province). *C. abbreviatus* is distributed in north Asia, Korea and most provinces of China [6]. It seems that there is nearly no geographical overlap between these two species and geographical information can be used to assign individuals to species correctly most of the time. However, incompatibility occurred when using external morphological characters to identify the specimens. Generally, the length of tegmina is the main character distinguishing these two species from each other. *C. italicus* has tegmina reaching or exceeding the apices of the hind femora, but *C. abbreviatus* has tegmina much shorter and distinctly not reaching the apices of the hind femora [6]. When examining specimens from all over China, we found that most specimens from localities south of theYangtze River had their tegmina obviously not reaching the apices of the hind femora and can be assigned to *C. abbreviatus* without any doubt. However, those from localities north of the Yangtze River had their tegmina reaching or distinctly exceeding the apices of the hind femora, and should be assigned to *C. italicus*. Therefore, either the distribution range of *C. italicus* should be extended to most provinces of north China from Xinjiang according to the morphological identification, or the taxonomic status of *C. abbreviatus* represented by populations in north China should be supported by additional evidence to clarify the incompatibility between the implications of morphological identification and geographical distribution ranges.

For the remaining two groups, the minor morphological differences between species within each group have usually been considered as normal variations among individuals from different geographical populations.

In this study, the overlap range between intraspecific variation and interspecific divergence was analyzed, the success rate of species identification was tested using “best match (BM)”, “best close match (BCM)”, “all species barcodes (ASB)” and “back-propagation neural networks (BP-based method)”, and species boundary delimitations of the eight species group were discussed based on the COI barcode sequences.

## Materials and Methods

### Taxon sampling

A total of 466 sequences from different individuals representing 4 superfamilies 5 families 49 genera and 72 species/subspecies were analyzed. Of these sequences, 20 were downloaded from GenBank (http://www.ncbi.nlm.nih.gov/) (Table S1), either COI fragments completely overlapped with the 658-bp Folmer region [75] or extracted from the published complete mitochondrial genome. At least five individuals from each population and as many populations as possible of the widespread species were sampled whenever the specimens were available (Table S2). However, sometimes only one or two individuals per species (or locality) could be collected. All specimens were preserved in 100% ethanol and stored at room temperature.

### DNA extraction, PCR amplification and sequencing

Whole genomic DNA was extracted from muscle tissue of the hind femur using a routine phenol/chloroform method [76]. Using the degenerated primer pair designed for Orthoptera [67], COBU (5′-TYTCAACAAAYCAYAARGATATTGG-3′) and COBL (5′-TAAACTTCWGGRTGWCCAAARAATCA-3′), the 658-bp fragments were amplified from the 5′ region of the cytochrome *c* oxidase 1 (COI) gene that has been adopted as the standard barcode for members of the animal kingdom [44].

The 25 µl PCR reaction mixture contained 13.875 µl of ultrapure water, 2.5 µl of 10× PCR buffer (Mg^2+^ free), 2.5 µl of MgCl_2_ (25 mM), 2 µl of dNTP (2.5 mM), 1.5 µl of each primer (0.01 mM), 0.125 µl of TaKaRa r-Taq polymerase, and 1 µl of DNA template. Amplifications were performed using a TaKaRa PCR Thermal Cycler. The cycling protocol consisted of an initial denaturation step at 95°C for 5 min, followed by 30–35 cycles of denaturation at 94°C for 45 s, annealing at 48°C for 45 s and extension at 70°C for 1 min 30 s, and a final extension at 72°C for 10 min and then held at 4°C. PCR products were used directly for sequencing after purification. Sequencing primers were the same as those for PCR amplification. Products were labeled using the BigDye® Terminator v.3.1 Cycle Sequencing Kit (Applied Biosystems, Inc.) and sequenced bidirectionally using an ABI PRISM™ 3100-Avant Genetic Analyzer.

### Sequence assembly and alignment

The raw sequence files were assembled using the Staden Package [77]. The assembled sequences were aligned using Clustal X [78], and both ends of the sequences matching the primer sequences were excised to remove artificial nucleotide similarity introduced by PCR amplification, resulting in the final length of 658 bp for both phylogenetic and barcoding analysis. COI nucleotide sequences were translated to amino acid sequences to check for stop codons and shifts in reading frame that might indicate the presence of nuclear mitochondrial copies (numts) [68]. Haplotype nucleotide sequences are deposited in GenBank (KC139803—KC140101, Table S3). Each haplotype was blasted using MEGABLAST option against the nucleotide collection (nr/nt), available on the NCBI website (http://blast.ncbi.nlm.nih.gov/Blast.cgi?CMD=Web&PAGE_TYPE=BlastHome). Only haplotypes that blasted within the correct suborder with E-values≤1.00E-30 were included in this study [53].

### Phylogeny reconstruction

To test the resolution of COI barcode sequences in reconstructing the phylogeny of grasshoppers at different taxonomic levels, we performed analyses in both parsimony and Bayesian frameworks with the following 4 species as outgroups: *Yunnanites coriacea* and *Atractomorpha sinensis* (Pyrgomorphoidea), *Bennia multispinata* (Eumastacoidea) and *Criotettix bispinosus* (Tetrigoidea).

Within the parsimony framework, the aligned sequence data were analyzed by using heuristic search algorithms with 100 random addition replicates implemented in PAUP 4.0 beta 10 win [79]. To assess support, we calculated standard bootstrap values based on 100 replicates (100 random-addition TBR replicates each).

Within the Bayesian framework, we analyzed the data sets by using the program MrBayes 3.1.2 [80] after selecting best-fit models of nucleotide evolution under the AIC criteria by using MrModeltest 3.7 [81]. The analyses consisted of running four simultaneous chains for 20 million generations (TVM+I+G), sampling every 1000 generations. Two independent identical Bayesian runs were performed to ensure convergence on similar results and the nodal support was assessed by using the posterior probability generated from a consensus tree of the trees sampled after burn-in. The amount of burn-in is the number of samples that will be discarded at the start of the run. Tracer 1.5 (http://beast.bio.ed.ac.uk) was used to view the point where the chain reached stationary and then the burn-in was determined by the number of samples before this point.

To provide a profile for the setup of taxa and groups for calculating genetic distances, a neighbor-joining (NJ) tree of K2P distances was created to provide a graphic representation of the patterning of divergence between species [82] because of its strong track record in the analysis of large species assemblages [83]. NJ tree building with 1000 bootstrap replicates was implemented in MEGA4.1 [84].

### Network analysis

Traditional bifurcating trees are less powerful for resolving relationship among intraspecific populations and closely related species than haplotype networks which can provide significant inferences about evolutionary relationships [85]–[86]. Therefore, we constructed haplotype networks for the eight aforementioned species groups. The construction of haplotype networks was implemented in TSC1.21 [87].

### Intraspecific variation, interspecific divergence and DNA barcoding overlap

Sequence divergences were calculated using the Kimura two parameter (K2P) distance model [88]–[89]. The calculation of the sequence divergences was implemented in MEGA4.1 [84]. From the sequence divergence data, the extent of DNA barcoding gap/overlap was then explored as typically done in barcoding studies [1].

### Species assignment with distance-based methods and the neural network approach

Distance-based methods of species assignments in conjunction with computer simulations are capable of determining the statistical significance of species identification success rates. We therefore performed the “best match (BM)” and “best close match (BCM)” [90] for the species with more than three individuals sampled, utilizing “single-sequence-omission” or “leave-one-out” simulation. The BCM identification protocol first identifies the best barcode match of a query, but only assigns the species name of that barcode to the query if the barcode is sufficiently similar. This approach requires a threshold similarity value that defines how similar a barcode match needs to be before it can be identified. Such a value could be estimated for a given data set by obtaining a frequency distribution of all intraspecific pairwise distances and determining the threshold distance below which 95% of all intraspecific distances are found. After the BCM analysis, a more rigorous application of the best close match strategy, all species barcodes (ASB), was implemented. Here information from all intraspecies barcodes in the reference data set was utilized instead of just focusing on the barcode that is most similar to the query. A list of all barcodes sorted by similarity to the query using the same threshold as for BCM was assembled for each query. Queries were considered a success when they were followed by all intraspecies barcodes as long as there were at least two barcodes for the species. With this approach, the identifier will be more confident about assigning this species name to the query than in cases where multiple species names are found on the list of best matches. Indeed, a conservative identifier would probably only assign a species name if the query is followed by all known barcodes for a particular species and insist that there are at least two intraspecies matches. The BM, BCM and ASB approaches are implemented in the computer program TaxonDNA (available at http://taxondna.sf.net/) [90].

Taking both accuracy and precision into account, we calculated an *ad hoc* distance threshold for our data set using a simple linear regression [91]. The query results were subdivided into (1) true positives (TP), (2) false positives (FP), (3) true negatives (TN), and (4) false negatives (FN) [91]. The performances of BCM were quantified by calculating accuracy ((TP+TN)/total number of queries) and precision (TP/(number of not-discarded queries)) as well as the overall identification (ID) error ((FP+FN)/total number of queries) and the relative ID error (FP/number of not-discarded queries). Variations in the proportions of TP, TN, FP, FN, accuracy, precision, overall and relative ID errors were quantified for 30 arbitrary K2P distance thresholds (THR_K2P_) ranging from THR_K2P_ = the largest query-best match K2P distance in a library (all queries are accepted as being correctly identified, i.e. none is discarded as in the BM criterion) to THR_K2P_ = 0.00 (only identical sequences are accepted as being correctly identified, all the others are discarded). Relationships between relative ID errors and K2P distance thresholds were investigated through linear regression. The regression equation was then used to infer an *ad hoc* distance threshold corresponding to the K2P distance yielding a relative ID error<0.05 (THR_K2P_0.05_). This *ad hoc* threshold corresponds to the K2P distance at which 95% of the not-discarded queries are expected to be correctly identified. The analysis was performed by Dr. Gontran Sonet using a script in R by himself.

Back-propagation neural network-based species identification (BP-based method) is a recently proposed method for DNA barcoding [92]–[93]. The BP-based method proved to be powerful in species assignments via DNA sequences, especially for closely related species [93]. We performed BP analysis for the same data set as we did BCM analysis. The data set was randomly divided into a reference data set and a query data set. The reference data set was used to train a BP-neural network model, while the query data set was used as a test data set. The ratio of reference sequences to query sequences was about 1∶1 since increasing the reference sequences did not significantly improve species identification success rate for the COI barcode [60]. For all these simulations, the learning rate was set to 0.2, moment value to 0.5 and training goal to 0.00001, as implemented in the program BPSI2.0 [92].

## Results

### Phylogeny reconstruction

**Figure 1:**
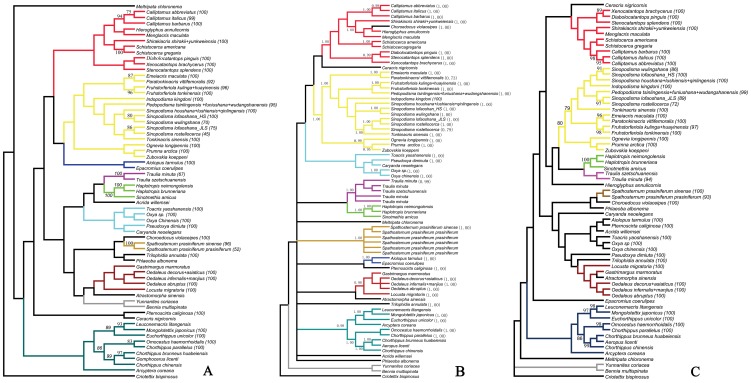
Phylogenetic analysis based on mtDNA COI in parsimony, Bayesian and neighbour-joining (K2P) frameworks. Subclades within a species with more than one individual sampledare collapsed. The number in parentheses indicates its bootstrap support values or posterior probabilities. Colored blocks of branches indicate the clades consistently recovered in parsimony, Bayesian and NJ analyses. Bootstrap values of <75 and posterior probability of <0.95 are not recorded on the tree. A. Cladogram from parsimony analysis. B. Cladogram from Bayesian analysis. C. Cladogram from NJ analysis.

Both parsimony and Bayesian analyses of our data set consistently recovered several monophyletic clades above genus levels (Fig. 1A, 1B). However, the relationships among these clades could not be resolved well, and none of the Catantopidae, Oedipodidae, Arcypteridae or Gomphoceridae were supported as monophyletic (Fig. S1). This result is consistent with earlier studies [13]–[15].

Excepting the *Spathosternum* and *Calliptamus* groups, each of the species groups we focused on was comprised of species that did not form reciprocally monophyletic clades. However, each of the six species groups usually formed a monophyletic clade most having high posterior probability and/or bootstrap values. In addition, *Sinopodisma lofaoshana* formed two distantly separated clades, with the clade of individuals from Jiulianshan population as a sister to the *S. rostellocerca* clade, and the clade of individuals from Hengshan population as a sister to the *S. wulingshana* clade. *Traulia minuta* included the single representative of *T. szetschuanensis* in the Bayesian tree but excluded it in the MP tree with a bootstrap value of less than 75%. Monophyletic clades of *Paratonkinacris vittifemoralis* were recovered both within the Bayesian and parsimony frameworks with posterior probability of 0.73 and bootstrap support of 92%.

Congeneric species formed monophyletic clades most of time in the case that two or more species were sampled from a genus. The exceptions were *Sinopodisma* into which the genus *Tonkinacris* fell, and *Chorthippus* which is a very difficult large grasshopper genus and needs a comprehensive revision (Fig. 1A, 1B). The genus *Pedopodisma* was recovered either as a sister to the *Sinopodisma*+*Tonkinacris* clade in the MP tree or as a sister to the *Fruhstorferiola* clade in the Bayesian tree.

The NJ analysis recovered a topology similar to those from parsimony and Bayesian analyses, with small differences in placements of a few species (Fig. 1C). All of the eight species groups we focused on formed monophyletic clades respectively, but individuals within each group, excluding groups of *Spathosternum* and *Calliptamus*, clustered neither by species nor populations (See the section on species boundary delimitation for the detailed clades).

To explore the further implications of the COI barcode fragment in reconstructing phylogeny at higher-levels, the data set was reanalyzed twice under MP framework with exclusion of third base data or both first and third base data. The results suggested that it seemed to have a little improvement in resolving higher-level relationships (Figs. S1, S2, S3). Although the higher-level relationships in the topologies were still not strongly supported and the monophylies of Catantopidae, Oedipodidae, Arcypteridae and Gomphoceridae were still not completely supported, most members of Catantopidae and Oedipodidae did show a closer relationship within family than between families (Figs. S2, S3).

### Intraspecific variation, interspecific divergence and DNA barcoding gap

Based on the neighbor-joining (NJ) tree of K2P distances, taxa or groups were set up to calculate the intraspecific variations and interspecific divergences. The results showed that variations within population were mostly distinctly less or slightly larger than 1%, intraspecific variations between populations were usually less than 3% (Table S4), a putative threshold for species assignment proposed by previous study [44], with *Sinopodisma lofaoshana* as the single exception which had much higher intraspecific variation (5.06–5.56%, average 5.25%) between interpopulation individuals. Two populations of *S. lofaoshana*, one from Hengshan and the other from Jiulianshan, were sampled; the variations within population were less than 1% but those between populations were slightly higher than 5%. Consequently, *S. lofaoshana* was divided into two groups to calculate intergroup divergences. The results showed that the divergence between Jiulianshan populations of *S. lofaoshana* and *S. rostellocerca* was as low as 2.17–3.12% (average 2.53%), indicating a closer relationship between them than between the two groups of *S. lofaoshana*.

**Figure 2:**
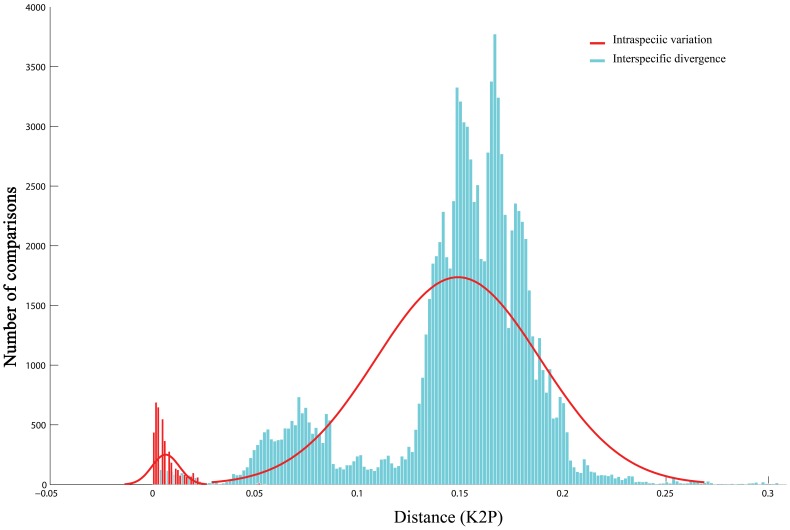
Distribution pattern of intraspecific variation and interspecific divergence.

The interspecific divergences within each of the six species groups abovementioned (Table S4) and 22.5% of interspecific divergences within a genus were less than 3%, 77.5% of them more than 3%, and 68.19% of them more than 5% (Table S5), resulting in a total overlap of 5.55% (from 0.0% to 5.55%) between intraspecific variations and interspecific divergences (Fig. 2).

### Species assignment through BCM and BP-based methods

In the case of identification with the BCM method, we assembled a data set including 432 sequences belonging to 43 species, with each species including at least three sequences to meet the requirement for “all species barcodes (ASB)” analysis. The threshold value for the identification simulation was calculated from the data set. Using the 95^th^ percentile of intraspecific distances (2%) computed from pairwise summary as the threshold for the BCM simulation, the correct identification according to “best close match (BCM)” is 91.66%, but that according to “all species barcodes (ASB)” is only 60.64% (Table 1). The 5.32% ambiguous and 2.77% incorrect identifications in BCM were all caused by the sequences from the six questionable species groups (Table S6). However, the much higher ambiguous identification in ASB analysis (38.88%) occurred also in query sequences of *Sinopodisma lofaoshana* and *S. rostellocerca* as well as in those of the six questionable species groups.

**Table 1:** Success rate of identification through “Best Match”, “Best Close Match” and “All Species Barcode”.

Identification method	Best Match	Best Close Match	All Species Barcodes
Correct identifications	397 (91.89%)	396 (91.66%)	262 (60.64%)
Ambiguous identifications	23 (5.32%)	23 (5.32%)	168 (38.88%)
Incorrect identifications	12 (2.77%)	12 (2.77%)	1 (0.23%)
Sequences without any match closer than 2.0%	----	1 (0.23%)	1 (0.23%)

**Figure 3:**
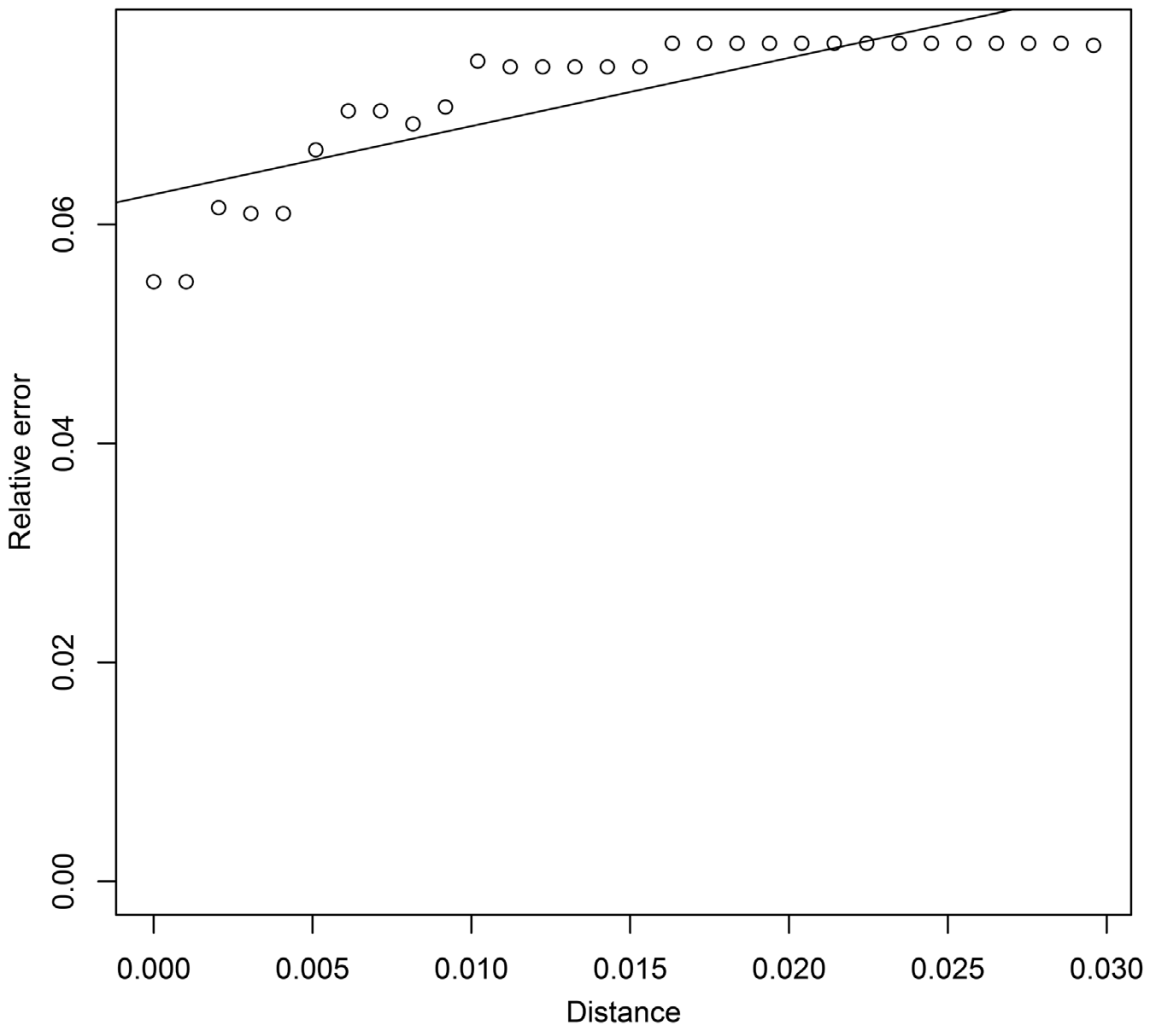
Relative identification errors at thirty arbitrary distance thresholds in BCM analysis.

**Figure 4:**
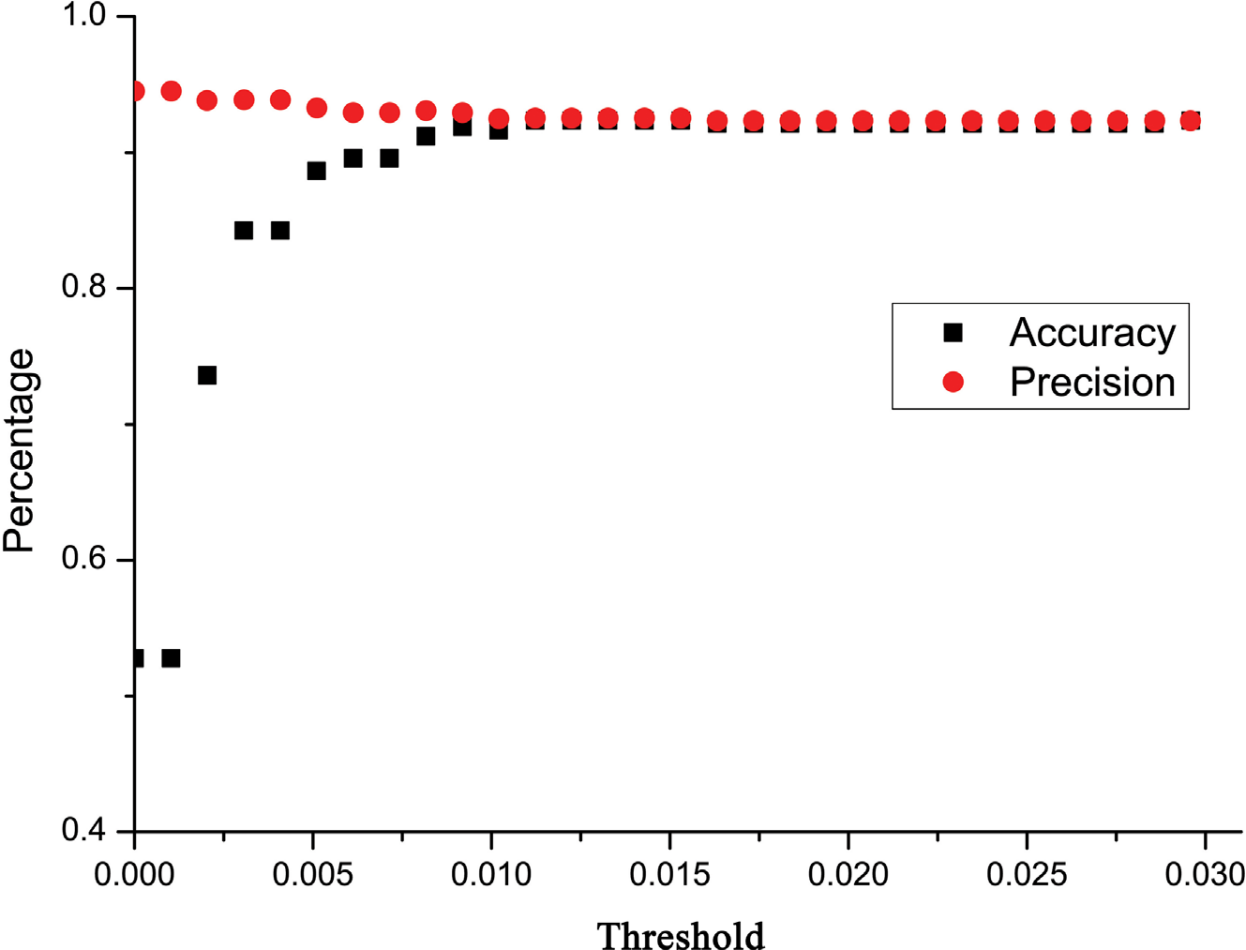
Accuracy and precision of identification at thirty distance thresholds ranging from K2P = 0.0295 to K2P = 0.000 in BCM analysis.

Taking both accuracy and precision into account, we calculated an *ad hoc* distance threshold for the data set using a simple linear regression [91]. Unfortunately, according to the analysis, we could not obtain an estimated relative identification error lower than 0.06 with the current data set (Fig. 3). According to the regression line, we would have to use a distance threshold of −0.02 to obtain an estimated relative identification error of 0.05, but a negative threshold is impossible to apply. By applying a threshold distance of 0, we can get the lowest estimated relative identification error possible with this data set, i.e. the intercept = 0.06. More restrictive distance thresholds will improve precision to some extent, yet negatively affect accuracy due to the higher proportions of queries discarded [91]. The use of more restrictive distance thresholds in our data set scarcely improved precision, but heavily decreased the accuracy when the threshold used was less than 0.01 (Fig. 4).

Instead of using the leave-one-out simulation for BCM methods, we used randomly selected reference and query sequences for BP-based method to investigate the performance of the barcode. This strategy was employed due to the slow training process which hinders the utility of the BP-based method in large scale simulation studies. Using the same sequence data as in BCM analysis, we assembled a training data set with 230 barcode sequences and a query data set with 202 barcode sequences. As a result, 10 query sequences were identified incorrectly with extremely high probability, 1 identified incorrectly with low probability, and 3 identified correctly with low probability, resulting in a success rate of 94.55%. All incorrect or inaccurate identification occurred in the six questionable species groups (Table S7).

### Species boundary delimitations for selected species groups

#### *Calliptamus italicus* species group

**Figure 5:**
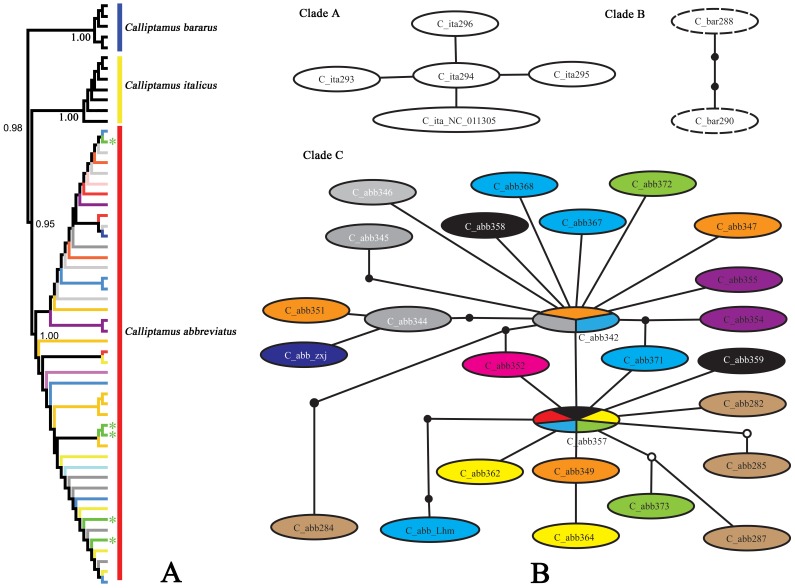
NJ tree subclade and haplotype network of three *Calliptamus* species. A. NJ tree subclade. Different colors of the edges indicate individuals from different populations, the green asterisks indicate the individuals from Xunhua county, Qinghai Province. B. Haplotype network. Different colors indicate haplotypes from different populations, the ovals filled with multiple colors indicate haplotypes shared by several populations, and the green color represents haplotypes from Qinghai Province.

Since there were some doubts on the relationship between *Calliptamus italicus* and *C. abbreviatus*, we first assigned a species name to each sequence according to the localities of materials under study, i.e. the materials from Xinjiang and Qinghai were provisionally considered as *C. italicus*, and those from other areas of China were considered as *C. abbreviatus*. The results showed that *C. bararus* and individuals of *C. italicus* from Xinjiang and Europe (NC_011305) formed monophyletic clades in the NJ tree respectively, both with 100% bootstrap value. However, the individuals of *C. italicus* from Qinghai did not cluster with those from Xinjiang and Europe, but formed another monophyletic clade with the specimens of *C. abbreviatus*. Individuals in the clade of *C. abbreviatus* did not cluster by populations (Fig. 5A).

Analysis with haplotype network led to a similar result. The network was divided into three separate clades, Clade A, Clade B and Clade C (Fig. 5B). Clade A was composed of haplotypes of *C. italicus* from Xinjiang and Europe, Clade B consisted of haplotypes of *C. barbarus*. However, the haplotypes of *C. italicus* from Qinghai (marked with green color) formed clade C together with those of *C. abbreviatus*, suggesting that the individuals sampled from Qinghai in this study actually belong to *C. abbreviatus*. Nearly no haplotypes of *C. abbreviatus* from the same population formed a relatively independent subclade, and two haplotypes were found shared by multiple populations, one shared by three populations (Siping, Jilin Province; Tongliao, Inner Mongolia Autonomous Region; Qin'an, Gansu Province) and the other by five populations (Anji, Zhejiang Province; Zhuolu, Hebei Province; Zhidan, Shaanxi Province; Qin'an, Gansu Province; Xunhua, Qinghai Province) (Fig. 5B).

Therefore, it is clear that *C. italicus* and *C. abbreviatus* are two separate species. However, the tegmina length should not be regarded as distinguishing character between these two species since wing-length polymorphism is quite common in Acrididae [94]–[95]. As for the distribution of *C. italicus* in Qinghai, study with larger scale sampling is needed to clarify it. Possibly, *C. italicus* is prevented from extending its distribution to Qinghai by the barrier of the Altai Mountains and Qilian Mountains. It is also impossible for *C. italicus* to spread from Xinjiang to neighboring provinces such as Gansu and Inner Mongolia because of the barriers imposed by the Altai Mountains and deserts.

### *Sinopodisma houshana* and *Pedopodisma tsinlingensis* groups

The species are extremely similar within each group. Nearly no morphological difference can be found among the species within each group except that the body size of *Sinopodisma houshana* is slightly larger than those of *S. lushiensis* and *S. qinlingensis*. All materials were collected from the type locality of each species except for those of *S. houshana*, which has a much more widespread distribution than *S. lushiensis* and *S. qinlingensis*. Therefore, there is no problem in assigning a morphospecies name to sampled individuals.

**Figure 6:**
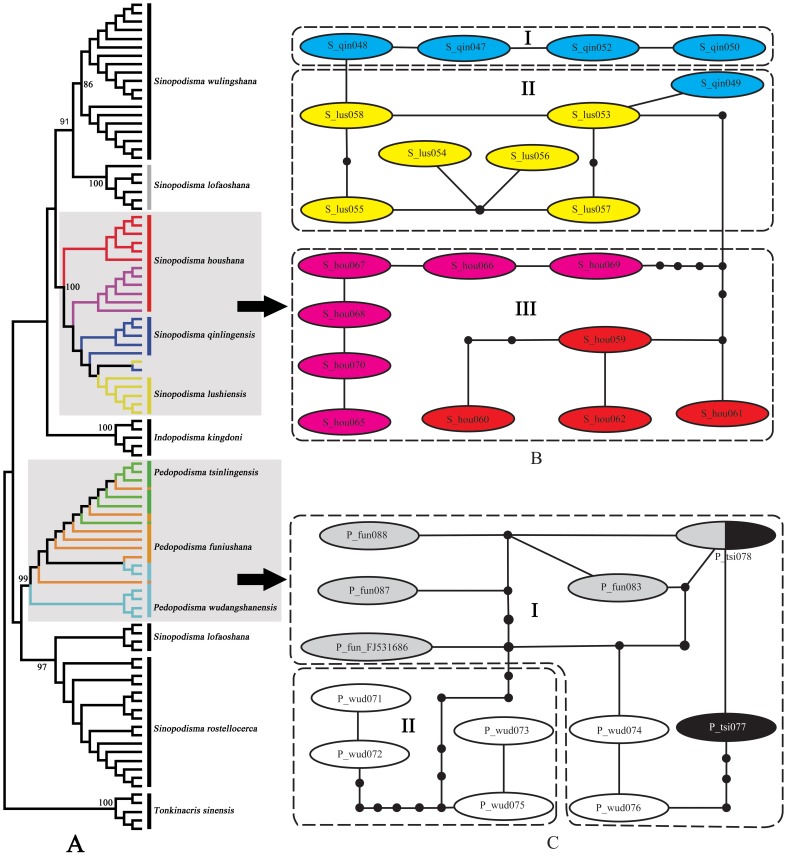
NJ tree subclade and haplotype network of the genera *Sinopodisma* and *Pedopodisma*. A. NJ tree subclade. B. Haplotype network of the *Sinopodisma houshana* species group; red color indicates Yinshan population and pink indicates Mulanshan population of *S. houshana*. C. Haplotype network of the *Pedopodisma tsinlingensis* species group.

The topology of the NJ tree showed that species of *S. houshana* group were slightly structured genetically but no species formed independent reciprocally-monophyletic clades (Fig. 6A). The intraspecific variations and interspecific divergences formed a complete overlap, with the latter falling completely within the range of the former (Table 2). The relationship among species within the *Pedopodisma tsinlingensis* group was even more confused; sequences of the three species scattered messily in the NJ tree and no species showed conspicuous genetic structure. The intraspecific genetic variations and interspecific divergences formed a broad overlap of 2.17% (Table 3).

**Table 2:** Intraspecific variation and interspecific divergence of the *Sinopodisma houshana* group.

Species	Intraspecific variation	Interspecific divergence	
		*S. houshana*	*S. lushiensis*
*S. houshana*	0∼2.17%		
*S. lushiensis*	0.15∼0.61%	0.77∼1.85%	
*S. qinlingensis*	0∼0.61%	0.92∼1.70%	0.15∼1.07%

**Table 3:** Intraspecific variation and interspecific divergence of the *Pedopodisma tsinlingensis* group.

Species	Intraspecific variation	Interspecific divergence	
		*P. tsinlingensis*	*P. funiushana*
*P. tsinlingensis*	0∼0.15%		
*P. funiushana*	0∼0.76%	0∼0.92%	
*S. wudangshanensis*	0.15∼2.17%	0.61∼2.33	0.46∼2.33%

In the networks, haplotypes of each group formed a separate clade (Fig. 6B, 6C). The clade of *S. houshana* group can be divided into three subclades, with subclade I composed of haplotypes of *S. qinlingensis*, subclade II composed of haplotypes of *S. lushiensis* and one of *S. qinlingensis*, and subclade III composed of haplotypes of *S. houshana*. Haplotypes of *S. lushiensis* had a minimum of one mutational step to those of *S. qinlingensis* and at least five mutational steps to those of *S. houshana*, indicating the much closer relationship of this species with *S. qinlingensis* than with *S. houshana*. The haplotypes of *S. houshana* formed a so-called independent subclade, the haplotypes from Yingshan population have a minimum of five mutational steps and those from Mulanshan population have a minimum of six mutational steps to those of *S. lushiensis*. However, there was a minimum of seven mutational steps between haplotypes from Yinshan and Mulanshan populations, indicating that both of them have a much closer relationship to *S. lushiensis* than between themselves. The clade of the *Pedopodisma tsinlingensis* group can be divided into two subclades, with subclade I containing haplotypes from all three species and subclade II containing haplotypes only from *P. wudangshanensis*. One haplotype was found shared by *P. tsinlingensis* and *P. funiushana*. Haplotypes of *P. wudangshanensis* in subclade I had a minimum of three mutational steps to *P. funiushana* and a minimum of four mutational steps to *P. tsinlingensis*, but had a minimum of nine mutational steps to those of the conspecifics in subclade II.

Therefore, the validities of *S. lushiensis*, *S. qinlingensis*, *P. funiushana* and *P. wudangshanensis* are questionable. It is reasonable to consider each group as a single species since they have no conspicuous difference in either morphological or molecular characters.

### *Fruhstorferiola kulinga* species group

**Figure 7:**
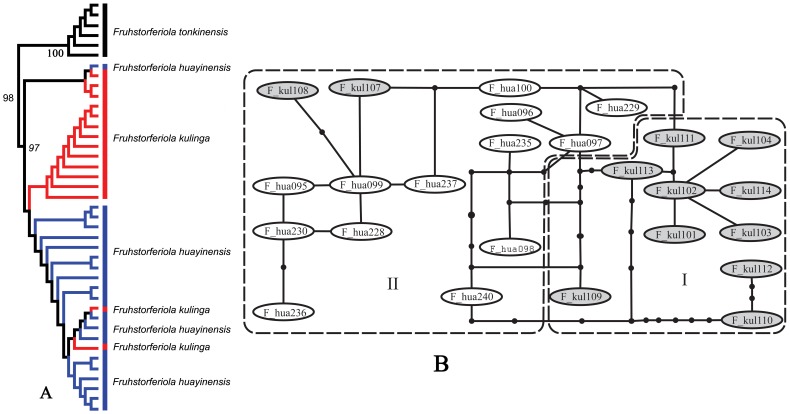
NJ tree Subclade and haplotype network of the genus *Fruhstorferiola*. A. NJ tree subclade. B. Haplotype network of the *Fruhstorferiola kulinga* group. F_kul indicates *Fruhstorferiola kulinga* (filled with grey color), F_hua indicates *Fruhstroferiola huayinensis*.

*Fruhstorferiola kulinga* and *F. huayinensis* are extremely similar to each other, and the characters used to distinguish them have been found to be substantially variable even within populations, so geographical distribution was used as the main criterion for preliminary identification of the materials under study. In the NJ tree, the sampled individuals of these two species displayed weak genetic structure to some extent, but did not form reciprocally monophyletic clades, with one individual of *F. huayinensis* falling into the clade of *F. kulinga* and two individuals of *F. kulinga* falling into the clade of *F. huayinensis* (Fig. 7A). The intraspecific variation of *F. kulinga* and *F. huayinensis* were 0∼2.97% and 0∼1.85% respectively, but the pairwise distances between the two species are 0.15∼2.96%, leading to a complete overlap, i.e. the interspecific divergences fell completely within the range of intraspecific variation of *F. kulinga*. The haplotype network of these two species can be divided into two subclades, with subclade I containing only haplotypes from *F. kulinga* and subclade II containing mainly haplotypes from *F. huayingensis* but two from *F. kulinga* (Fig. 7B).

Since the present molecular data do not separate these two species from each other, and the morphological characters used to distinguish them have been found to be unstable, we would like to treat them provisionally as a single species before further phylogeographical research with much larger scale samples.

### *Shirakiacris shirakii* species group

**Figure 8:**
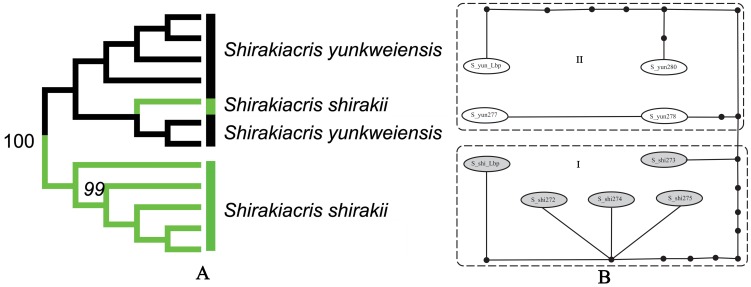
NJ tree subclade and haplotype network of the *Shirakiacris shirakii* group. A. NJ tree subclade. B. Haplotype network.

There are different opinions on the validity of *Shirakiacris yunkweiensis* [6], [70]. In this study, the limited samples provided additional evidence on the relationship between these two species. In the NJ tree (Fig. 8A), one individual of *S. shirakii* fell into the clade of *S. yunkweiensis*. The ranges of intraspecific variation within *S. shirakii* and *S. yunkweiensis* were 0∼1.7% and 0∼1.54% respectively, and the interspecific pairwise distances were 0.61∼2.01%, leading to a overlap of as much as 1.09% between intraspecific variation and interspecific divergence. The haplotype network can be divided into two subclades (Fig. 8B), with subclade I containing haplotypes from *S. shirakii* and subclade II containing haplotypes from *S. yunkweiensis*, but one haplotype of *S. shirakii* had a minimum of four mutational steps to *S. yunkweiensis*, much smaller than the minimum of ten mutational steps to other haplotypes of *S. shirakii*.

Since no results from any analysis supported the validity of *S. yunkweiensis*, it should be regarded as a junior synonym of *S. shirakii* [70].

### *Spathosternum prasiniferum* species group

**Figure 9:**
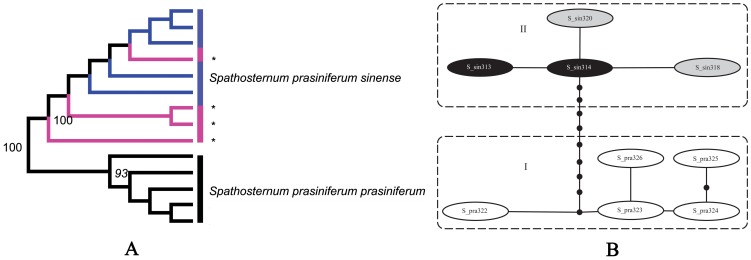
NJ tree subclade and haplotype network of the *Spathosternum prasiniferum* species group. A. NJ tree subclade. The asterisks and pink color indicate the female individuals of *S. prasiniferum sinense* from Guilin with long tegmina. B. Haplotype network. S_pra represents *S. prasiniferum prasiniferum* and S_sin represents *S. prasiniferum sinense*. Black color indicates the haplotypes from the normal individuals of *S. prasiniferum sinense* with reduced tegmina. Grey color indicates the haplotypes from the female individuals of *S. prasiniferum sinense* from Guilin with long tegmina.

While *Spathosternum prasiniferum sinense* and *S. p. prasiniferum* are regarded at present as two subspecies of the same species, they formed reciprocally monophyletic clades in the NJ tree (Fig. 9A) and there was a conspicuous gap of 0.93% (from 0.61% to 1.54%) between intraspecific variations (0∼0.3% and 0.15∼0.61% respectively; Table S4) and interspecific divergences (pairwise distances varying between 1.54∼1.85%). The haplotype network displayed a similar relationship and there was a minimum of ten mutational steps between the nominal subspecies (Fig. 9B). Interestingly, there were four female individuals with long tegmina collected together with *S. p. sinense* from Guilin. Judged solely according to morphology, these four females should be recognized as *S. p. prasiniferum*, but they formed a monophyletic clade in the gene tree with the short-winged individuals from the same population (Fig. 9A). The pairwise distances between these four long-winged individuals and short-winged individuals varied between 0∼0.3%, indicating that they were much closer genetically to and may be aberrant individuals of *S. p. sinense*. Furthermore, there were haplotypes shared by long- and short-winged individuals from the same population.

Therefore, *S. p. sinense* and *S. p. prasiniferum* are at least two distinct subspecies since they have conspicuous morphological differences and molecular divergences. Considering the common presence of polymorphism in wing length in Acrididae [94]–[95], the relatively low genetic distances between the two subspecies (1.54∼1.85%) and the broad distribution of *S. prasiniferum*, data from more populations will certainly facilitate making a categorical decision. Possibly, the taxonomic status of *S. p. sinense* could even be raised to species-level based on futher evidences from phylogeographical study.

### *Oedaleus decorus* and *Oedaleus infernalis* species groups

**Figure 10:**
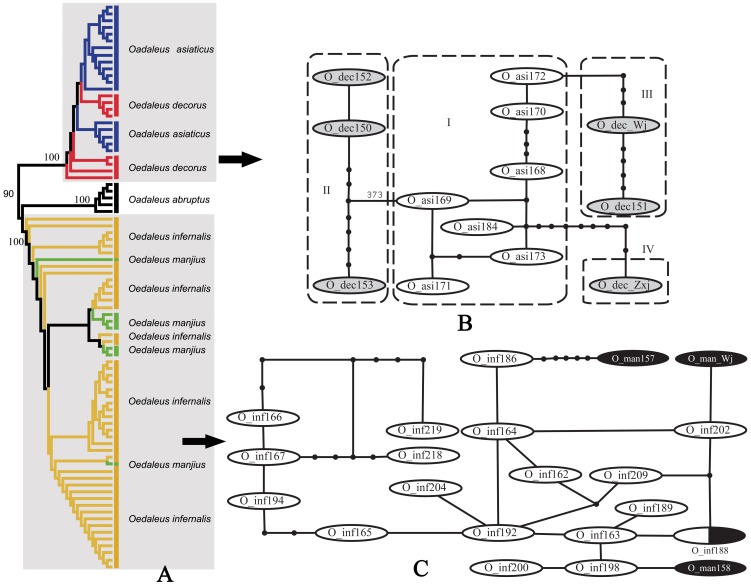
NJ tree subclade and haplotype network of *Oedaleus*. A. NJ tree subclade. B. Haplotype network of the *Oedaleus decorus* group. O_dec indicates *O. decorus* and O_asi indicates *O. asiaticus*. C. Haplotype network of the *Oedaleus infernalis* group. O_inf indicates *O. infernalis* and O_man indicates *O. manjius*, the oval filled with white-black color indicates the haplotype shared by *O. infernalis* and *O. manjius*.

*Oedaleus decorus* differs from *O. asiaticus* mainly in having the dark transverse band of hind wing broader and not interrupted at the first anal vein. Eight individuals of *O. decorus* from three populations of Xinjiang and Gansu and eighteen individuals of *O. asiaticus* from six populations were sampled in this study. They did not form reciprocally monophyletic clades in the NJ tree (Fig. 10A). The ranges of intraspecific variations within *O. decorus* and *O. asiaticus* were 0∼2.17% and 0∼1.23% respectively, and the interspecific pairwise distances were 1.39∼2.01%, leading to a complete overlap between intraspecific variations and interspecific divergences. The haplotypes of *O. decorus* were connected to the central network of *O. asiaticus* through three subclades, and there was a minimum of four mutational steps between the haplotypes of the two species (Fig. 10B).

*Oedaleus infernalis* can be distinguished from *O. manjius* mainly by its yellowish brown tibiae and the narrower dark transverse band of the hind wing. Forty-four individuals of *O. infernalis* from 11 populations and seven individuals of *O. manjius* from 2 populations were sampled in this study. However, they did not form reciprocally monophyletic clades in the NJ tree (Fig. 10A). The intraspecific variations of *O. infernalis* and *O. manjius* ranged from 0 to 2.47% and 0 to 1.7% respectively, and the interspecific pairwise distances from 0 to 2.63%, leading to a broad overlap between intraspecific variations and interspecific divergences within this group. The haplotypes of *O. manjius* were connected to the network of *O. infernalis* through three subclades, and one haplotype was found shared by them (Fig. 10C).

Therefore, the minor morphological differences between the nominal species within each group may be attributed to adaptation to different environments and each group should be actually considered as comprising a single widely-distributed species. Further phylogeographical study may reveal much more precise genetic structure pattern in these two groups, including the color pattern polymorphism that commonly exist in Acrididae [94], [96].

## Discussion

### Performance of COI barcode sequence in reconstructing higher-level phylogeny

Although the primary aim of our study was to investigate DNA barcoding, the phylogeny inferred from COI barcode sequence sheds new light on the relationship among taxa within Acrididae. Given that the genus *Pedopodisma* did not fall within *Sinopodisma* either in the MP tree or in the Bayesian tree, it likely be an independent genus [6] rather than a junior synonym of *Sinopodisma* [71]. Further study with thorough sampling will certainly facilitate a better understanding of the relationship between *Sinopodisma* and *Pedopodisma*. Since the monophylies of most genera and well delineated species were supported by our data set, it seemed that COI barcode region performed much better in phylogenetic reconstruction at genus and species levels than at higher levels. It is reasonable to assume that the success of species assignment may be higher where the reconstructed evolution of the gene reflects speciation events, particularly where closely related species are under study [97].

Although the phylogeny at higher levels were not well resolved using COI barcode sequence, a little improvement was gained when the third base data or both first and third base data were excluded. This limited improvement may be due to: (1) the fact that some groups may be paraphyletic or polyphyletic; (2) the bias in taxon sampling; and (3) the use of the partial fragment of the single COI gene. It is expected that combining COI barcode data with other gene sequences (especially suitable nuclear genes), more thorough sampling and the exploration of new analytical strategies will certainly improve our understanding of the phylogeny of Acridoidea at higher-levels.

### Efficacy of DNA barcoding in Acridoidea

The most important goal of DNA barcoding is to facilitate the species discovery process by increasing the speed, objectivity, and efficiency of species identification. However, barcoding failure associated with non-monophyly is very likely at the traditionally recognized species level [1]. Such a mismatch of taxonomy and genealogy was also observed in the New Zealand grasshopper genus *Sigaus* [68]. In our data set, when the traditional species names were used, interspecific divergences extended at their lower end well into the range of intraspecific variation (Fig. 2). Species assignment through each of the “best close match (BCM)”, “all species barcodes (ASB)” and “BP-based neural network” methods produced error rates of more than 5% (Tables 1). Since nearly all incorrect identifications occurred in the sequences of the six questionable species groups (Table S6, S7), and the species boundary delimitation supported our presumption on the relationships among species within each species group, this error rate appeared to be mainly a result of imperfect taxonomy. Furthermore, no *ad hoc* threshold was available to obtain an estimated relative identification error of 0.05. This result might be due to the high proportion of false positives caused by too high genetic similarities among extremely close species. Such a high genetic similarity can derive from either incomplete lineage sorting among newly diverged species or imperfect taxonomy. Therefore, it is expected that a much higher success rate could be obtained when the revised species names are applied to the data set. It is obvious that perfect taxonomy will greatly increase the success rate of identification through DNA barcoding, and that taxonomic revision plays an important role in DNA barcoding studies [1].

The single representative of *Traulia szetschuanensis* was resolved within *Traulia minuta* in Bayesian analysis (Fig. 1B) but outside of the latter in both the MP tree (Fig. 1A) and the NJ tree (Fig. 1C). It is possible that *T. szetschuanensis* will form a monophyletic clade in phylogenetic trees when more individuals are sampled. The divergence between them was 4%, much higher than the threshold of 3% and the 95^th^ percentile distance threshold (2%), indicating that they can be correctly identified through DNA barcoding.

While *Tonkinacris sinensis* fell into the genus *Sinopodisma* in both of the MP and Bayesian analyses, it was recovered as a monophyletic clade that was the sister of the clade of *Sinopodisma rostellocerca*+*S. lofaoshana* (Jiulianshan population) (Fig. 1A, 1B). It fell outside of *Sinopodisma* in the NJ tree (Fig. 1C) and had an interspecific divergence of 5.89% from *Sinopodisma rostellocerca* and of 6.48% from the Jiulianshan population of *Sinopodisma lofaoshana*. That is to say, *Tonkinacris sinensis* can be correctly identified under a completely sampled phylogeny through DNA barcoding using either tree-based or threshold or other species identification approaches.

**Figure 11:**
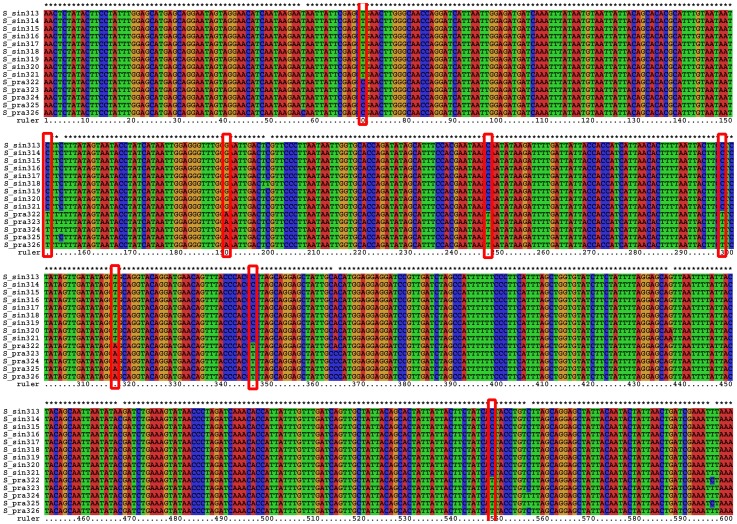
Alignment of *Spathosternum*, showing the purely diagnostic positions (position 70, 151, 190, 247, 298, 316, 346, 548, marked with red boxes) and the total variable positions. S_sin313∼321 indicate sequences of *S. prasiniferum sinense* (including the four long-winged individuals from the Guilin population)and S_pra322∼326 indicate *S. prasiniferum prasiniferum*.

While the average interspecific divergence between *Spathosternum prasiniferum prasiniferum* and *S. p. sinense* is only 1.7%, they were reciprocally monophyletic in the MP and NJ trees, and both have much constrained intraspecific variation (Table S4), giving rise to a distinctive gap between intraspecific variation and interspecific divergence. Furthermore, eight purely diagnostic positions, nearly half of the total variable positions were found within the barcode sequence alignment (Fig. 11), and bases private to one of the two subspecies existed in all the other variable positions, suggesting a conspicuous genetic divergence between these two taxa which may be very recently diverged species. Therefore, molecular data derived from much more extensive sampling should serve as evidence to revive the species status of *S. sinense* and they can be correctly identified most of time by using either morphological characters (the length of tegmina) or the COI barcoding fragment through a thoroughly sampled phylogeny. As for the four females of *S. p. sinense* with long tegmina from Guilin, their similarity to *S. p. prasiniferum* in tegmen length may be a result induced by environmental factors. Such aberrant individuals occur extremely rarely and have nearly no genetic difference from the abundant normal individuals with short tegmina. In such a case, it is DNA barcoding but not a traditional morphological approach that provides a more accurate identification for the aberrant individuals. Of course, geographical information will help us in identifying such individuals if further study can support their allopatry. While the intraspecific variation of *Sinopodisma lofaoshana* exceeded the interspecific divergences of *S. p. prasiniferum* against *S. p. sinense*, they were in different parts of the tree. Although having a substantial impact during the discovery phase (i.e., in an incompletely sampled group), such overlap will not affect identification of unknowns in a thoroughly sampled tree [1].

*Sinopodisma lofaoshana* is the most difficult taxon in our data set. Two populations were sampled in this study and the result showed that the intraspecific variations between individuals of different populations (5.06–5.56%, average 5.25%) distinctly overlapped with the interspecific divergence between individuals of Jiulianshan population of *S. lofaoshana* and those of *S. rostellocerca* (2.17–3.12%, average 2.53%). This overlap did not lead to any incorrect or ambiguous identification in either BCM or BP-based analysis because each population has more than one individual sampled and the intraspecific variation within population was below 1% (0–0.92%). However, the ambiguous identification occurred when performing the ASB simulation because of the presence of the genetic distance overlap between these two species, suggesting that ASB, as a more rigorous barcoding method, can reveal much more sensitively the presence of a possible taxonomic problem in some groups. Both *S. lofaoshana* and *S. rostellocerca* have widespread distributions in south China and appear to have an overlap zone. Although both populations of *S lofaoshana* formed monophyletic clades, further study with thorough sampling is needed to reveal the geographical genetic structures and to clarify its relationship to *S. rostellocerca*.

Nearly all erroneous identifications in the BCM and BP-based analyses were caused by the six questionable species groups abovementioned. If it can be confirmed by further evidence that inappropriate morphological classification does exist in these groups, then a comprehensive revision, i.e. the synonymy for each group based on morphology and supported by other evidence, will lead to more accurate identification through DNA barcoding. In any case, accurate taxon identification is extremely important for molecular studies [98].

In a word, the 91% of correct identification for the BCM method and the 94% for the BP-based method in Acridoidea are acceptable at the moment and consistent with the results reported in previous studies [58], [90]. While mtDNA barcoding cannot offer good resolution at higher taxonomic levels, the completeness of the DNA barcode database against which unknown sequences are compared will certainly increase the accuracy of species identification [99].

### Implications of DNA barcoding in species boundary delimitation

The accuracy of a threshold-based approach critically depends upon the level of overlap between intraspecific variation and interspecific divergence across a phylogeny. However, the ranges of both intraspecific variation and interspecific divergence will change in response to species delineation, thus leading to a variation of the overlap [1]. Since many traditional species will appear to be genetically non-monophyletic because of imperfect taxonomy [61], it is necessary to make a comprehensive revision of any group before or in combination with DNA barcoding study. Nearly all traditional species were established based only on morphological character sets, and many species, especially those described much earlier, were not compared with relevant type specimens of closely-related species when newly described. Thus all species, especially if they are closely-related, should firstly be revised to confirm the presence of morphological differences among them, and this will resolve directly to some extent the issue of oversplitting caused by the lack of type comparison [1].

In this study, the barcoding sequence data set not only tested the success rate of identification but also provided additional data for the morphologically questionable species groups. DNA barcoding supported our doubts about taxonomic accuracy in six species groups and the validity of species in two groups, and facilitated a better understanding of the relationship among species within each species group. Furthermore, ASB analysis also revealed the particular relationship between *Sinopodisma lofaoshana* and *S. rostellocerca*, which needs further study with more thorough sampling. Therefore, DNA barcoding can not only increase the speed and accuracy of species identification, but also help us highlight or resolve some taxonomic issues.

As an identification tool, DNA barcoding should be used in conjunction with other information [52]. Species with distinct morphological differences observed should still be corroborated with other sources of data, including geographical, biological, ecological, reproductive, behavioral and DNA sequence information [54]. If no additional non-morphological difference can be found among species established by morphology alone, the conclusion should be that only one species exists with morphological polymorphism, and synonymy should be proposed. Similarly, we should re-examine morphology or move on to some other source of information once fixed DNA differences are observed among aggregates in which morphological differences have not previously been observed. This may show whether the DNA sequence divergences represent truly morphologically cryptic species or genetic polymorphism within a single species. In other words, from the barcoding perspectives, substantial sequence differentiation can be explained as heteroplasmy or due to numts if it is not confirmed with other sources of information. Conversely, even as few as one nucleotide of consistent differentiation can serve as a diagnostic character to identify and delineate a valid species if it is distinctly different morphologically, or ecologically, or in other aspects from its closely related congeneric species [100]. Putative cryptic species detected by mtDNA barcoding merit closer investigation via analysis of, or more in-depth examination of, ecology and taxonomy [100]–[101]. Nuclear genetic data can be used as a check when mtDNA barcoding reveals unexpected results, because deep genetic divisions found during mtDNA barcoding are not always reflected in the nuclear genome in some groups [102]–[103].

In conclusion, despite the problems of sampling size [104]–[105] and the criticisms on methodological [106], theoretical [107] and empirical grounds [1], [108]–[110], the prospect of DNA barcoding is still promising if it is based on solid foundations of comprehensive taxonomy. The exploration on new analytical methods [59], [93], [99], [105], [111]–[117] and the use of nuclear genes as additional effective DNA barcodes [60], [103] will certainly promote the progress in DNA barcoding.

## Supporting Information

Figure S1 **MP tree infferred with complete base data.** Members of Catantopidae are marked with red, those of Oedipodidae with deep blue, those of Arcypteridae with yellow, those of Gomphoceridae with pink, those of Acrididae with green, those of Pamphagidae with bright blue and other groups with black.
(TIF)

Figure S2 **MP tree infferred with exclusion of third base data.** Members of Catantopidae are marked with red, those of Oedipodidae with deep blue, those of Arcypteridae with yellow, those of Gomphoceridae with pink, those of Acrididae with green, those of Pamphagidae with bright blue and other groups with black.
(TIF)

Figure S3 **MP tree infferred with exclusion of both third and first base data.** Members of Catantopidae are marked with red, those of Oedipodidae with deep blue, those of Arcypteridae with yellow, those of Gomphoceridae with pink, those of Acrididae with green, those of Pamphagidae with bright blue and other groups with black.
(TIF)

Table S1 **Sequences downloaded from NCBI.**
(DOC)

Table S2 **Materials used in this study.**
(DOC)

Table S3 **Cross reference list between GenBank accession numbers and numbers of vouchers sharing the same haplotype.**
(DOC)

Table S4 **ranges of intraspecific genetic variations.**
(DOC)

Table S5 **Distribution of intraspecific variations and interspecific divergences.**
(DOC)

Table S6 **Sequences identified as ambiguous or incorrect in BCM analysis.**
(DOC)

Table S7 **Assignment of species through BP-based method.**
(DOC)
