# Supplementary figures and images for: DNA Barcoding and Species Boundary Delimitation of Selected Species of Chinese Acridoidea (Orthoptera: Caelifera)
Source: PLoS One. 2013 Dec 20;8(12):e82400. doi: 10.1371/journal.pone.0082400 (PMC3869712; doi:10.1371/journal.pone.0082400)

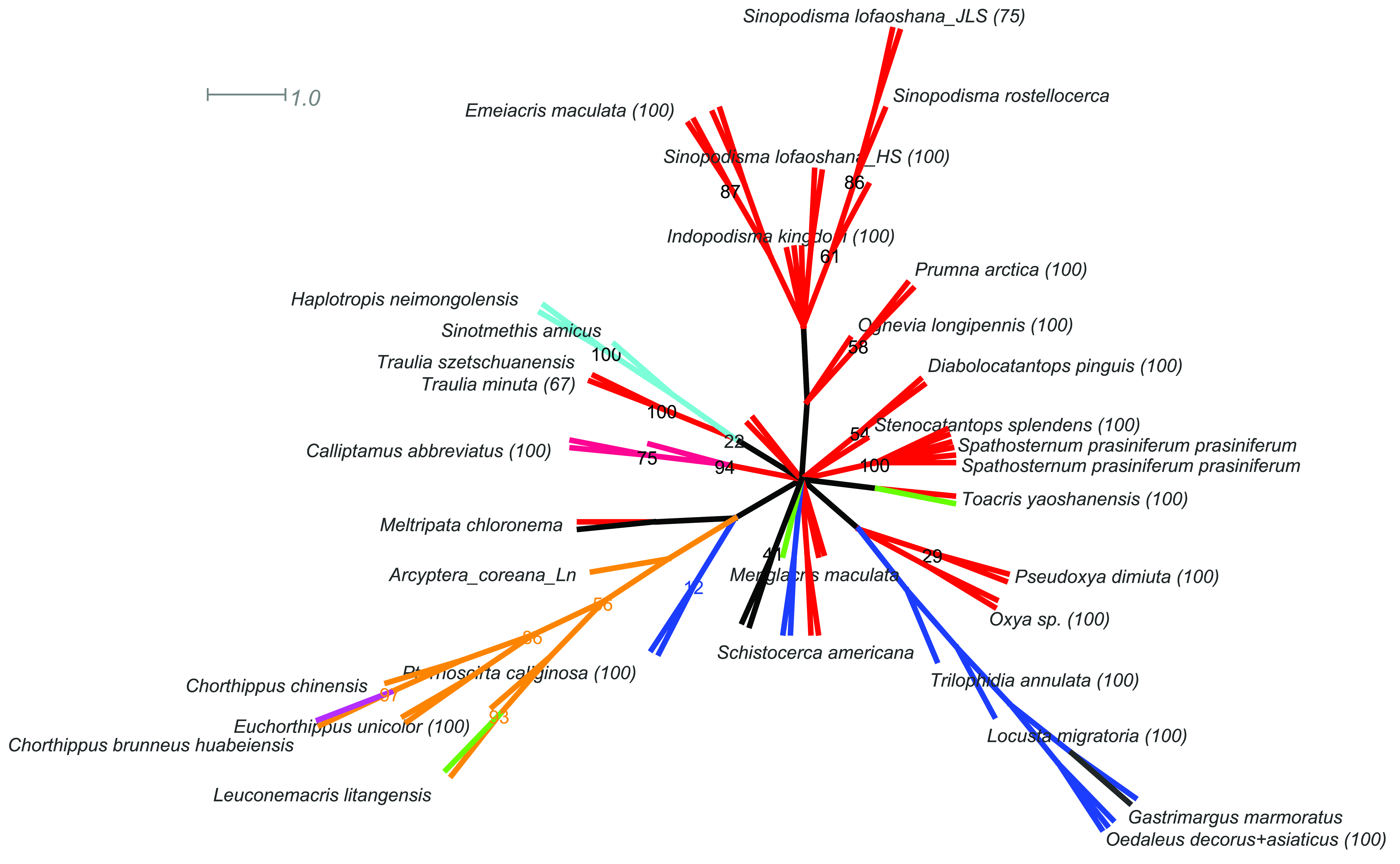

Supplement: Figure S1 — MP tree infferred with complete base data. Members of Catantopidae are marked with red, those of Oedipodidae with deep blue, those of Arcypteridae with yellow, those of Gomphoceridae with pink, those of Acrididae with green, those of Pamphagidae with bright blue and other groups with black. (TIF) [file pone.0082400.s001.tif]

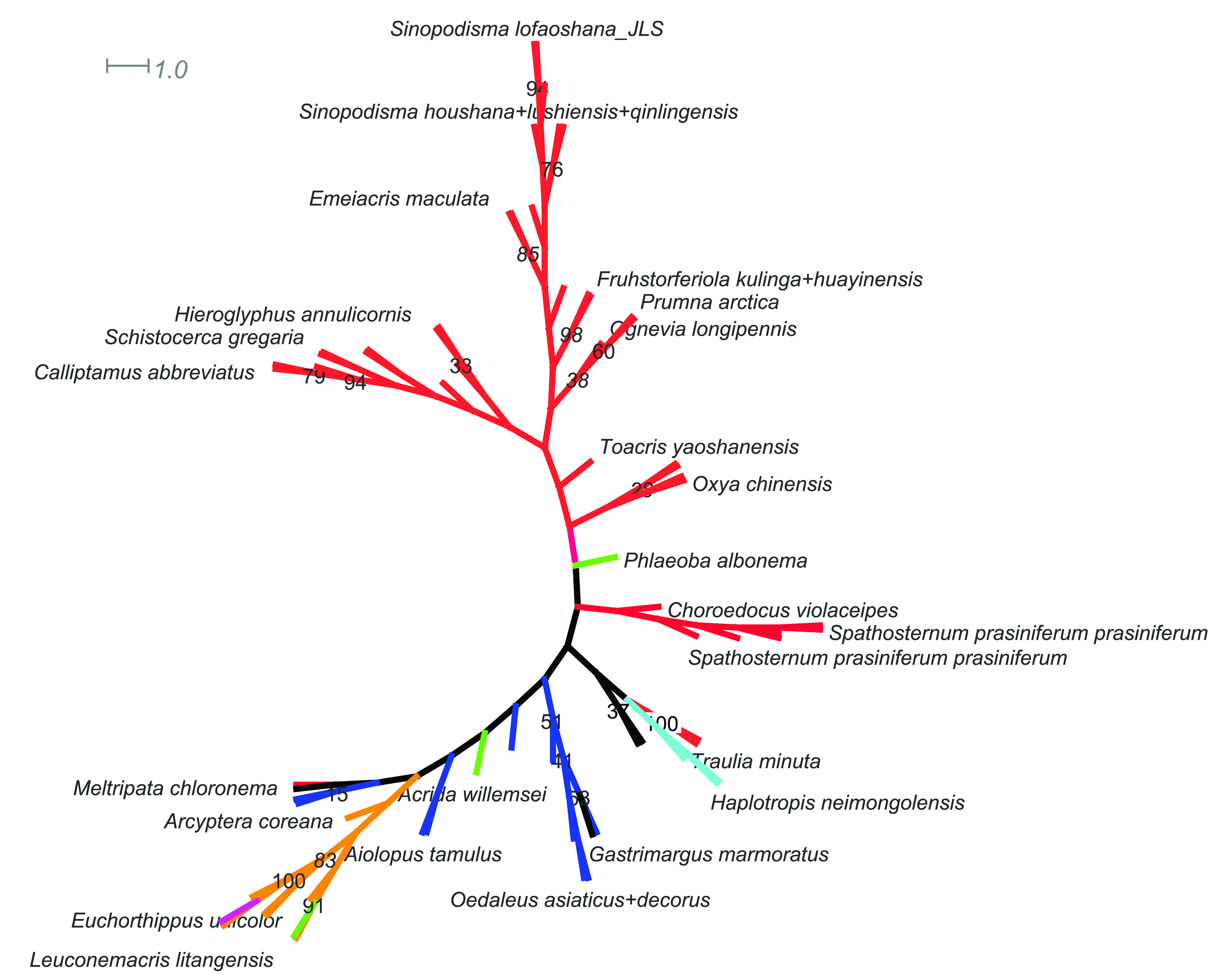

Supplement: Figure S2 — MP tree infferred with exclusion of third base data. Members of Catantopidae are marked with red, those of Oedipodidae with deep blue, those of Arcypteridae with yellow, those of Gomphoceridae with pink, those of Acrididae with green, those of Pamphagidae with bright blue and other groups with black. (TIF) [file pone.0082400.s002.tif]

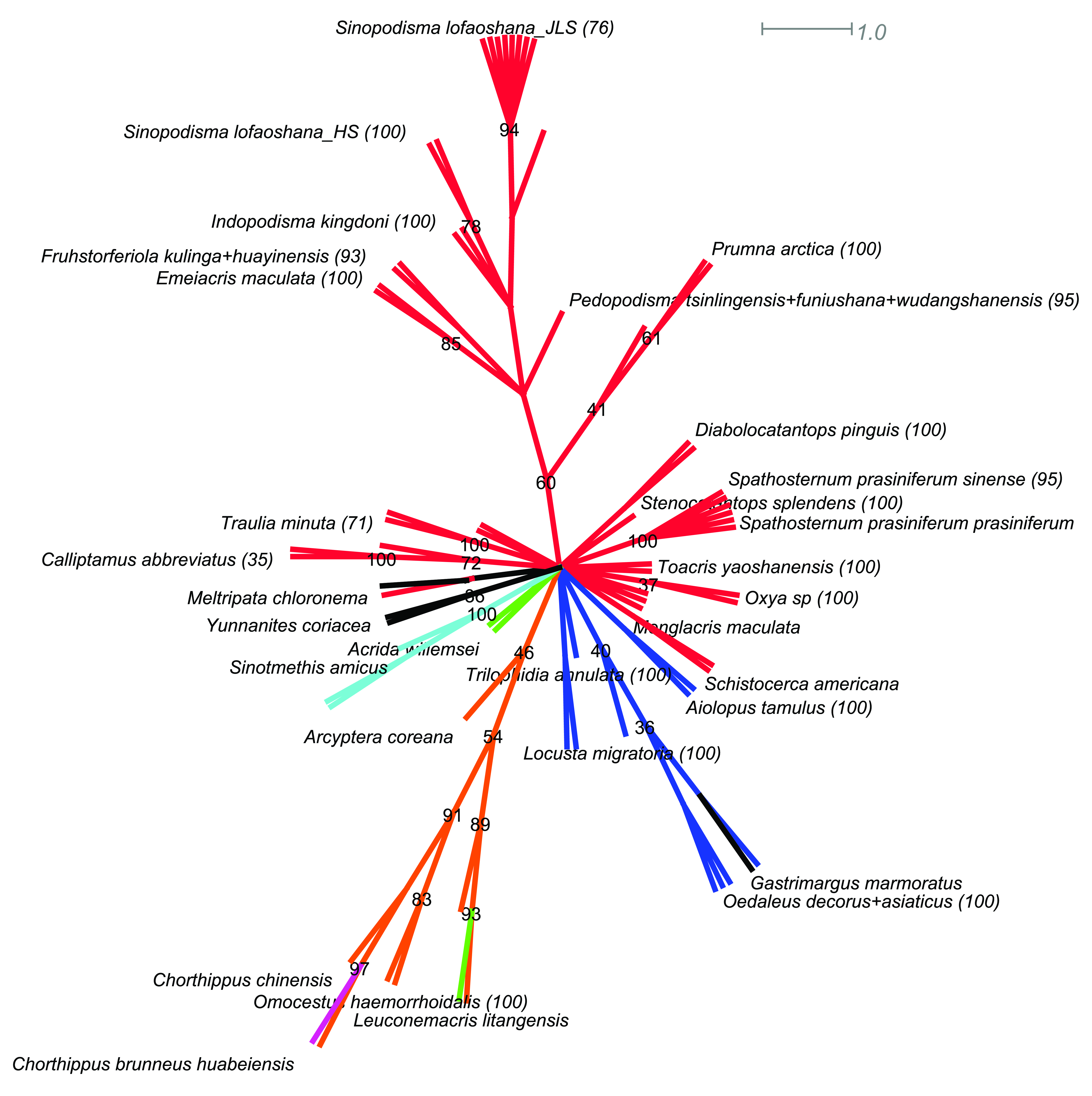

Supplement: Figure S3 — MP tree infferred with exclusion of both third and first base data. Members of Catantopidae are marked with red, those of Oedipodidae with deep blue, those of Arcypteridae with yellow, those of Gomphoceridae with pink, those of Acrididae with green, those of Pamphagidae with bright blue and other groups with black. (TIF) [file pone.0082400.s003.tif]
