# Supplementary material for: DNA Barcoding and Species Boundary Delimitation of Selected Species of Chinese Acridoidea (Orthoptera: Caelifera)
Source: PLoS One. 2013 Dec 20;8(12):e82400. doi: 10.1371/journal.pone.0082400 (PMC3869712; doi:10.1371/journal.pone.0082400)
Supplement: Table S1 — Sequences downloaded from NCBI. (DOC) [file pone.0082400.s004.doc]

**Table S1. Sequences downloaded from NCBI**

| Species | Locality | NCBI accession | Reference |
| --- | --- | --- | --- |
| *Calliptamus italicus* (**Calliptaminae**) | Brje pri Komnu, Slovenia | EU938373 ( NC_011305) | Fenn, *et al*, 2008 |
| *Traulia minuta* (**Coptacridinae**) | Mengla, Yunnan, China | FJ571149 | Zhao, *et al*, 2010 |
| *Traulia szetschuanensis* (**Coptacridinae**) | Neixiang, Yunnan, China | FJ571148 | Zhao, *et al*, 2010 |
| *Meltripata chloronema* (**Coptacridinae**) | Mengla, Yunnan, China | FJ571150 | Zhao, *et al*, 2010 |
| *Choroedocus violaceipes* (**Eyprepocnemidinae**) | Jinggong,Yunnan, China | FJ571158 | Zhao, *et al*, 2010 |
| *Pedopodisma funiushana* (**Melanoplinae**) | Lushi, Henan, China | FJ531686 | Zhao, *et al*, 2010 |
| *Prumna arctica* (**Melanoplinae**) | Tahe, Helongjiang | FJ531674 | Zhao, *et al*, 2010 |
| *Oxya chinensis* (**Oxyinae**) | Chang’an, Xi’an, Shaanxi, China; | NC_010219 | Zhang & Huang, 2007 |
| *Schistocerca americana* (**Cyrtacanthacridinae**) |  | EU589056 | Song, , *et al*, 2008. |
| *Schistocerca gregaria* (**Cyrtacanthacridinae**) |  | NC_013240 | Erler, *et al*, 2010 |
| *Gastrimargus marmoratus* (**Oedipodinae**) |  | NC_011114 | Ma, *et al*, 2009 |
| *Locusta migratoria* (**Oedipodinae**) |  | NC_001712 | Flook, *et al*, 1995 |
| *Locusta migratoria* *migratoria* (**Oedipodinae**) | Buerjin, Xinjiang, China | NC_011119 | Direct submission |
| *Oedaleus asiaticus* (**Oedipodinae**) |  | NC_011115 | Ma, et al, 2009 |
| *Chorthippus parallelus* (**Gomphocerinae**) |  | X95574, X95575 | Szymura, et al, 1996 |
| *Chorthippus chinensis* (**Gomphocerinae**) | Jiugongshan, Hubei, China | NC_011095 | Liu & Huang, 2008 |
| *Acrida willemsei* (**Acridinae**) | Malaysia, Sabah, Croker Rng. NP,  HQ station Rd. | EU938372 (NC_011303) | Fenn, et al, 2008 |
| *Phlaeoba albonema* (**Acridinae**) | Tianhetan, Guiyang, China | NC_011827 | Shi, et al, 2008 |
| *Atractomorpha sinensis* (**Pyrgomorphoidea**) |  | NC_011824 | Ding, et al, 2007 |

**References**

Ding FM, Shi HW, Huang Y (2007) Complete mitochondrial genome and secondary structures of lrRNA and srRNA of *Atractomorpha sinensis* (Orthoptera, Pyrgomorphidae). *Zoological Research*, 28：580－588

Erler S, Ferenz HJ, Moritz RFA, Kaatz HH (2010) Analysis of the mitochondrial genome of *Schistocerca gregaria gregaria* (Orthoptera: Acrididae). *Biological Journal of Linnaeus Society of London*, 99: 296—305.

Fenn JD, Song H, Cameron SL, *et al.* (2008) A preliminary mitochondrial genome phylogeny of Orthoptera (Insecta) and approaches to maximizing phylogenetic signal found within mitochondrial genome data. *Molecular Phylogenetics and Evolution*, 49: 59—68.

Flook PK, Rowell CH, Gellissen G (1995) The sequence, organization, and evolution of the *Locusta migratoria* mitochondrial genome. *Journal of Molecular Evolution,* 1995, **41:** 928—941.

Folmer O, Black M, Hoeh W, *et al*. (1994) DNA primers for amplification of mitochondrial cytochrome c oxidase subunit I from diverse metazoan invertebrates. *Molecular Marine Biology and Biotechnology*, 3: 294-297.

Liu Y, Huang Y (2008) Sequencing and analysis of complete mitochondrial genome of *Chorthippus chinensis* Tarb. *Chinese Journal of Biochemistry and Molecular Biology*, 24 (4): 329—335.

Ma C, Liu CX, Yang PC, *et al*. (2009) The complete mitochondrial genomes of two band-winged grasshoppers, *Gastrimargus marmoratus* and *Oedaleus asiaticus. BMC Genomics* 2009, **10**:156. doi:10.1186/1471-2164-10-156

Shi HW, Ding FM, Huang Y (2008) Complete Sequencing andAnalysis of mtDNA in *Phlaeoba albonema* Zheng. *Chinese Journal of Biochemistry and Molecular Biology*, 24 (7) :604～611

Song H, Buhay JE, Whiting MF, *et al*. (2008) Many species in one: DNA barcoding overestimates the number of species when nuclear mitochondrial pseudogenes are coamplified. *Proceedings of the National Academy of Science*, USA, 105(36): 13486—13491.

Szymura JM, Lunt DH, Hewitt GM (1996) The sequence and structure of the meadow grasshopper (Chorthippus parallelus) mitochondrial srRNA, ND2, COI, COII ATPase8 and 9 tRNA genes. *Insect Molecular Biology*, 5(2): 127—139.

Zhao L, Zheng ZM, Huang Y, *et al*. (2010) Phylogeny of the Chinese Cantatopidae (Orthopter : Acridoidea) inferred from mitochondrial DNA sequences. *Acta　Scientiarum Naturalium Universitatis Sunyatseni*, 49(4): 111—117.

Zhang CY, Huang Y (2007). Complete mitochondrial genome of *Oxya chinensis* (Orthoptera: Acridoidea). *Acta Biochimica et Biophysica Sinica*, 40, 7–18.
