# Supplementary material for: DNA Barcoding and Species Boundary Delimitation of Selected Species of Chinese Acridoidea (Orthoptera: Caelifera)
Source: PLoS One. 2013 Dec 20;8(12):e82400. doi: 10.1371/journal.pone.0082400 (PMC3869712; doi:10.1371/journal.pone.0082400)
Supplement: Table S2 — Materials used in this study. (DOC) [file pone.0082400.s005.doc]

**Table S2. Materials used in this study**

| Species | Voucher number | Locality, time and collector |
| --- | --- | --- |
| **Acridoidea: Acrididae: Melanoplinae** |  |  |
| *Sinopodisma rostellocerca* | gl0001-0002, 0014-0016 | Yaoshan, Guilin, Guangxi, China; 27 July 2006; Jianhua Huang |
|  | gl0003-0004, 0011-0013 | Yong’an, Xing’an County, Guangxi, China; 4 July 2006; Jianhua Huang |
|  | gl0005-0006 | Huangsha, Lingui County, Guangxi, China; 13 August 2006; Jianhua Huang |
|  | gl0007-0010 | Gaozhai, Xing’an County, Guangxi, China; 6 August 2006; Jianhua Huang |
| *Sinopodisma lofaoshana* | gl0017, 0019-0022 | Hengshan, Hunan, China; 28 August 2007; Jianhua Huang |
|  | gl0026 | Chukou, Zixing County, Hunan, China; 5 July 2005; Jianhua Huang |
|  | gl0023-0025, 0027 | Jiulianshan, Jiangxi, China; 21 July 2008; Fuming Shi |
| *Sinopodisma wulingshana* | gl0018, 0029-0034 | Hengshan, Hunan, China; 29 August 2007; Jianhua Huang |
|  | gl0035-0040 | Daweishan, Liuyang, Hunan, China; 5 October 2007; Jianhua Huang |
|  | gl0041-0046 | Xingdoushan, Lichuan, Hubei, China; 3 August 2004; Yulin Zhong |
| *Sinopodisma qinlingensis* | gl0047-0052 | Xunyangba, Ningshan, Shaanxi, China; 18 July 2008; Yuan Huang |
| *Sinopodisma lushiensis* | gl0053-0058 | Shiziping, Lushi, Henan, China; 25 September 2008; Jianhua Huang |
| *Sinopodisma houshana* | gl0059-0064 | Yingshan, Hubei, China; 26 August 2004; Yulin Zhong |
|  | gl0065-0070 | Mulanshan, Wuhan, Hubei, China; 29 September 2008; Jianhua Huang |
| *Pedopodisma wudangshanensis* | gl0071-0076 | Wudangshan, Hubei, China; 27 July 2004; Yulin Zhong |
| *Pedopodisma tsinlingensis* | gl0077-0082 | Nanwutai, Chang’an, Shaanxi, China; 7 September 2008; Jianhua Huang |
| *Pedopodisma funiushana* | gl0083-0088 | Shiziping, Lushi, Henan, China; 25 September 2008; Jianhua Huang |
| *Fruhstorferiola tonkinensis* | gl0089-0094 | Yong’an, Xing’an, Guangxi, China; 2 July 2006; Jianhua Huang |
| *Fruhstorferiola huayinensis* | gl0095-0100 | Haopingshi, Taibaishan, Shaanxi, China; 13 July 2005; Zhaoqiang Qian |
|  | gl0227-0231 | Nanwutai, Chang’an, Shaanxi, China; 7 September 2008; Jianhua Huang |
|  | gl0232-0234 | Huayangchuan, Huayin, Shaanxi, China; 3 September 2008; Jianhua Huang |
|  | gl0235-0240 | Baiyunshan, Songxian, Henan, China; 17 August 2008; Jianhua Huang |
| *Fruhstorferiola kulinga* | gl0101-0106 | Hengshan, Hunan, China; 29 August 2007; Jianhua Huang |
|  | gl0107-0108 | Jingshan, Jingzhou, Hubei, China; 10 October 2007; Fuming Shi |
|  | gl0109-0112 | Longmenhe, Xingshan, Hubei, China; 4 August 2003; Yulin Zhong |
|  | gl0113-0115 | Gaozhai, Xing’an, Guangxi, China; 9 August 2006; Jianhua Huang |
| *Emeiacris maculata* | gl0241-0246 | Hengshan, Hunan, China; 29 August 2008; Jianhua Huang |
| *Paratonkinacris vittifemoralis* | gl0247-0251 | Gaozhai, Xing’an, Guangxi, China; 8 July 2009; Jianhua Huang |
| *Ognevia longipennis* | gl0252-0256 | Yangjiaping, Zhuolu, Hebei, China; 20 August 2005; Yuan Huang |
|  | Zxj-4 | Qianshan, Liaoning, China; 3 August 2007; Huimin Sun |
|  | Zcy-2 | Yuxian, Hebei, China; 21 August 2005; Yuan Huang |
| *Tonkinacris sinensis* | gl0257-0261 | Yong’an, Xing’an, Guangxi, China; 2 July 2006; Jianhua Huang |
| *Prumna arctica* | gl0262-0266 | Shibazhan, Heilongjiang, China; 1 September 2007; Huimin Sun |
|  | Zxj-1 | Tahe, Heilongjiang, China; 1 September 2007; Xiaojing Zhang |
| *Indopodisma kingdoni* | gl0267-0271 | Chayu, Xizang, China; 1 October 2007; Fuming Shi |
| *Zubovskia koeppeni* | Qzy-M0709 | Mianduhe, Yakeshi, Inner Mongolia, China; 21 July 2007; collector unknown |
| **Eyprepocnemidinae** |  |  |
| *Shirakiacris shirakii* | gl0272, 0274-0276 | Chang’an, Shaanxi, China; 1 November 2003; Rongsheng Lu |
|  | gl0273 | Yaoshan, Guilin, Guangxi, China; 27 July 2007; Jianhua Huang |
|  | Lbp-LC12 | Chang’an, Shaanxi, China; 1 November, 2001; collector unknown |
| *Shirakiacris yunkweiensis* | gl0277-0281 | Xiachayu, Chayu, Xizang, China; 3 October 2007; Zhijun Zhou |
|  | Lbp-LC13 | Dali, Yunnan, China; 4 August 1997; collector unknown |
| *Choroedocus violaceipes* | Cch-M0806 | Puer, Yunnan, China; 31 July 2007; Yuan Huang |
| **Calliptaminae** |  |  |
| *Calliptamus abbreviatus* | gl0282-0287 | Songbai, Shennongjia, Hubei, China; 31 July 2004; Yulin Zhong |
|  | gl0342-0346 | Siping, Jilin, China; 1 August 2007; Wei Ye |
|  | gl0347-0351 | Daqinggou, Tongliao, Inner Mongolia, China; 1 August 2007; Wei Ye |
|  | gl0352 | Tieling, Liaoning, China; 2 August 2007; Wei Ye |
|  | gl0353 | Anji, Zhejiang, China; 10 July 2006; Fangmei Ding |
|  | gl0354-0356 | Xincheng, Shanxi, China; 15 October 2006; Chenyan Zhang |
|  | gl0357-0361 | Yangjiaping, Zhuolu, Hebei, China; 20 August 2005; Yuan Huang |
|  | gl0362-0366 | Zhidan County, Shaanxi, China; 15 September 2004; Gang Chang |
|  | gl0367-0371 | Qin’an County, Gansu, China; 6 August 2006; Yuan Huang |
|  | gl0372- gl0376 | Xunhua, Qinghai, China; 25 July 2004; Jing Hu |
|  | Lbp-LC17 | Qin’an County, Gansu, China; 8 August 1999; collector unknown |
|  | Zxj-8 | Jiagedaqi, Heilongjiang, China; 6 September 2007; Huimin Sun |
|  | Lhm-M0505 | Yongjing, Gansu, China; 8 August 2001; Yuan Huang |
| *Calliptamus barbarus* | gl0288-0292 | Xunhua, Qinghai, China; 25 July 2004; Jing Hu |
| *Calliptamus italicus* | gl0293- 0297 | Mulei County, Xinjiang, China; 15 August 2005; collector unknown |
|  | Zxj-9 | Mulei County, Xinjiang, China; 13 August 2005; Xiaojing Zhang |
| **Catantopinae** |  |  |
| *Diabolocatantops pinguis pinguis* | gl0298-0301 | Zhashui, Shaanxi, China; 8 September 2002; collector unknown |
|  | gl0302 | Jiangkou, Ningshan, Shaanxi, China; 13 September 2008; Jianhua Huang |
| *Stenocatantops splendens* | gl0303-0307 | Xiachayu, Chayu, Xizang, China; 3 October 2007; Zhijun Zhou |
| *Xenocatantops brachycerus* | gl0308-0312 | Huashan, Huayin, Shaanxi, China; 2 September 2008; Jianhua Huang |
|  | Lbp-LC14 | Zhashui, Shaanxi, China; 7 September 2002; collector unknown |
| **Spathosterninae** |  |  |
| *Spathosternum prasiniferum sinense* | gl0313-0317 | Yaoshan, Guilin, Guangxi, China; 27 July 2006; Jianhua Huang |
|  | gl0318-0321 | Yaoshan, Guilin, Guangxi, China; 27 July 2006; Jianhua Huang (with tegmina reaching apex of hind femur, 4♀♀ from the same population as gl0313-0317) |
| *Spathosternum prasiniferum prasiniferum* | gl0322-0326 | Jin’gu County, Yunnan, China; 31 July 2007; Yuan Huang |
| **Oxyinae** |  |  |
| *Toacris yaoshanensis* | gl0327-0331 | Dayaoshan, Jinxiu, Guangxi, China; 12 June 2006; Jianhua Huang |
| *Pseudoxya diminuta* | sl0318-0321 | Liuwanshan, Yulin, Guangxi, China; 3 August 2006; Jia Li |
|  | sl0322-0325 | Mengla, Yunnan, China; 31 July 2007; Fuming Shi |
| *Oxya sp.* | sl0330-0332 | Haikou, Hainan, China; 12 October 2003; Yuan Huang |
| *Oxya chinensis* | sl0333-0334 | Shiziping, Lushi, Henan, China; 20 August 2008; Jianhua Huang |
| *Caryanda neoelegans* | Cch-M0807 | Menglun, Yunnan, China; July 1998; Yuan Huang |
| **Coptacridinae** |  |  |
| *Traulia minuta* | sl0311-0315 | Caiyanghe, Simao, Yunnan, China; 28 July 2007; Yuan Huang |
|  | Lgd-02 | Caiyanghe, Simao, Yunnan, China; 28 July 2007; Yuan Huang |
| **Heiroglyphinae** |  |  |
| *Hieroglyphus annulicornis* | Cch-M0805 | Hangzhou, Zhejiang, China; July 2007; Fangmei Ding |
| **Habrocneminae** |  |  |
| *Menglacris maculata* | Qzy-M0705 | Caiyanghe, Puer, Yunnan, China; 28 July 2007; Yuan Huang |
| **Oedipodinae** |  |  |
| *Trilophidia annulata* | gl0116-0120 | Yangjiaping, Zhuolu, Hebei, China; 20 August 2005; Yuan Huang |
|  | gl0121 | Baoding, Hebei, China; 19 August 2005; Yuan Huang |
|  | gl0122-0126 | Tieling, Liaoning, China; 2 August 2007; Wei Ye |
|  | gl0127-0129 | Ya’an, Sichuan, China; 11 August 2007; Yuan Huang |
|  | gl0130-0131 | Qin’an, Gansu, China; 6 August 2006; Yuan Huang |
|  | gl0132-0137 | Jinan, Shandong, China; 23 September 2007; Yuan Huang |
|  | gl0138 | Jin’gu, Yunnan, China; 31 July 2007; Yuan Huang |
|  | gl0139-0143 | Menglun, Yunnan, China; 24 July 1998; Yuan Huang |
|  | gl0144-0145 | Lantian, Shaanxi, China; 23 September 2007; collector unknown |
|  | gl0146-0149 | Chang’an, Shaanxi, China; 2 November 2008; Jianhua Huang |
|  | Wj-HC03 | Weiqu, Chang’an, Shaanxi, China; 31 July 2002; Jing Wang |
| *Oedaleus decorus* | gl0150-0155 | Mulei, Xinjiang, China; 23 August 2005; collector unknown |
|  | Zxj-12 | Xinyuan, Xinjiang, China; 15 August 2006; Xin Li |
|  | Wj-HC05 | Su'nan, Gansu, China; 26 July 2001; Yuan Huang |
| *Oedaleus* *asiaticus* | gl0168-0173 | Xinbaerhuzuoqi, Inner Mongolia, China; 23 July 2007; Weigong Wang |
|  | gl0174 | Jinhekou, Yuxian, Hebei, China; 23 August 2005; collector unknown |
|  | gl0175-0179 | Qin’an, Gansu, China; 6 August 2006; Yuan Huang |
|  | gl0180-0183 | Xunhua, Qinhai, China; 25 July 2004; Jing Hu |
|  | gl0184 | Hulunbeier, Inner Mongolia; 26 July 2007; Chengying Pan |
| *Oedaleus manjius* | gl0156-0161 | Yong’an, Xing’an, Guangxi, China; 4 July 2006; Jianhua Huang |
|  | Wj-HC06 | Zhashui, Shaanxi, China; 17 September 2002; collector unknown |
| *Oedaleus infernalis* | gl0162-0167 | Qin’an, Gansu, China; 6 August 2006; Yuan Huang |
|  | gl0185-0186 | Zhashui, Shaanxi, China; 18 September 2002; collector unknown |
|  | gl0187 | Duqu, Chang’an, Shaanxi, China; 13 September 2003; collector unknown |
|  | gl0188-0190 | Xunyangba, Ningshan, Shaanxi, China; 19 September 2002; collector unknown |
|  | gl0191-0196 | Yangjiaping, Zhuolu, Hebei, China; 20 August 2005; Yuan Huang |
|  | gl0197-0202 | Jinan, Shandong, China; 23 September 2007; Yuan Huang |
|  | gl0203-0208 | Alukeerqian, Chifeng, Inner Mengolia, China; 3 August 2007; Wei Ye |
|  | gl0209-0214 | Siping, Jilin, China; 1 August 2007; Wei Ye |
|  | gl0215-0220 | Tieling, Liaoning, China; 3 August 2007; Wei Ye |
|  | Wj-HC04 | Linxia, Gansu, China; 12 August 2001; Yuan Huang |
|  | Zxj-11a | Dunhua, Jilin, China; 12 August 2007; Huimin Sun |
|  | Zxj-11b | Dunhua, Jilin, China; 12 August 2007; Xiaojing Zhang |
| *Oedaleus abruptus* | gl0337-0341 | Sanlidian, Guilin, Guangxi, China; 17 September 2009; Jianhua Huang |
| *Aiolopus tamulus* | gl0221-0226 | Chang’an, Shaanxi, China; 2 November 2008; Jianhua Huang |
|  | gl0332-0336 | Sanlidian, Guilin, Guangxi, China; 17 September 2009; Jianhua Huang |
|  | Wj-HCO2 | Haikou, Hainan, China; 12 October 2003; Yuan Huang |
| *Pternoscirta caliginosa* | sl0326 | Jigongshan, Xinyang, Henan, China; 7 August 2009; Shaoli Mao |
|  | sl0327-0329 | Jiangkou, Ningshan, Shaanxi, China; 13 September 2008; Jianhua Huang |
| *Epacromius coerulipes* | Wsz-4 | Qin’an, Guansu, China; May 2006; Yuan Huang |
| *Locusta migratoria* *manilensis* | Xll-2 | Hubei, China; 5 August 2002; Yulin Zhong |
| **Gomphocerinae** |  |  |
| *Ceracris nigricornis* | Xll-M0631 | Shennongjia, Hubei, China; 19 August 2004; Yulin Zhong |
| *Omocestus haemorrhoidalis* | sl0335-0339 | Huayangchuan, Huayin, Shaanxi, China; 3 September 2008; Jianhua Huang |
| *Euchorthippus unicolor* | sl0340-0344 | Huayangchuan, Huayin, Shaanxi, China; 3 September 2008; Jianhua Huang |
| *Arcyptera coreana* | Ln-M0501 | Taibaishan, Meixian, Shaanxi, China; 17 July 2002; Nian Liu |
| *Leuconemacris litangensis* | Gj-M0802 | Litang, Sichuan, China; 9 August 2007; Yuan Huang |
| *Chorthippus brunneus huabeiensis* | Zxj-17 | Beian, Heilongjiang, China; 27 August 2007; Huimin Sun |
| *Aeropus licenti* | Gj-M0803 | Inner Mongolia, China; 19 July 2007; collector unknown |
| *Mongolotettix japonicus* | Zxj-30 | Wudalianchi, Heilongjiang, China; 25 August 2007; Xin Li |
|  | Ly-2 | Yuxian, Hebei, China; 21 August 2005; Yunlin Zhong |
| **Pamphagidae** |  |  |
| *Haplotropis neimongolensis* | Zxj-29 | Jiagedaqi, Heilongjiang, China; 6 September 2007; Huimin Sun |
| *Haplotropis brunneriana* | Hj-M0502 | Taibaishan, Meixian, Shaanxi, China; 17 July 2007; Jing Hu |
| *Sinotmethis amicus* | Gj-M0801 | Wenquan, Xinjiang, China; 24 July 2005; collector unknown |
| **Pyrgomorphoidea** |  |  |
| *Yunnanites coriacea* | Xll-M0632 | Mengla, Yunnan, China; 21 July 2004; collector unknown |
| **Eumastacoidea** |  |  |
| *Bennia multispinata* | Zxj-39 | Yunnan, China; 21 July 2004; collector unknown |
| **Tetrigoidea** |  |  |
| *Criotettix bispinosus* | Wsz-1 | Beihai, Guangxi, China; February 2005; Shizhen Wei |
