# Supplementary material for: DNA Barcoding and Species Boundary Delimitation of Selected Species of Chinese Acridoidea (Orthoptera: Caelifera)
Source: PLoS One. 2013 Dec 20;8(12):e82400. doi: 10.1371/journal.pone.0082400 (PMC3869712; doi:10.1371/journal.pone.0082400)
Supplement: Table S3 — Cross reference list between GenBank accession numbers and numbers of vouchers sharing the same haplotype. (DOC) [file pone.0082400.s006.doc]

**Table S3** **Cross reference list between GenBank accession numbers and numbers of vouchers sharing the same haplotype**

| **GenBank accession number** | **number of vouchers sharing the same haplotype** | **GenBank accession number** | **number of vouchers sharing the same haplotype** |
| --- | --- | --- | --- |
| KC139803 | gl0282, gl0283, gl0286 | KC139953 | gl0032 |
| KC139804 | gl0284 | KC139954 | gl0035 |
| KC139805 | gl0285 | KC139955 | gl0036 |
| KC139806 | gl0287 | KC139956 | gl0037 |
| KC139807 | gl0342, gl0343, gl0348, gl0350, Lbp-LC17 | KC139957 | gl0038 |
| KC139808 | gl0344 | KC139958 | gl0039 |
| KC139809 | gl0345 | KC139959 | gl0l040 |
| KC139810 | gl0346 | KC139960 | gl0041 |
| KC139811 | gl0347 | KC139961 | gl0042 |
| KC139812 | gl0349 | KC139962 | gl0043 |
| KC139813 | gl0351 | KC139963 | gl0044 |
| KC139814 | gl0352 | KC139964 | gl0045 |
| KC139815 | gl0354, gl0356 | KC139965 | gl0046 |
| KC139816 | gl0355 | KC139966 | gl0262 |
| KC139817 | gl0353, gl0357, gl0360, gl0361, gl0363, gl0365, gl0366, gl0370, gl0374, gl0375 | KC139967 | gl0263 |
| KC139818 | gl0358 | KC139968 | gl0264 |
| KC139819 | gl0359 | KC139969 | gl0265 |
| KC139820 | gl0362 | KC139970 | gl0266 |
| KC139821 | gl0364 | KC139971 | Zxj-1 |
| KC139822 | gl0367 | KC139972 | gl0257, gl0260 |
| KC139823 | gl0368, gl0369 | KC139973 | gl0258, gl0259, gl0261 |
| KC139824 | gl0371 | KC139974 | Qzy-M0709 |
| KC139825 | gl0372 | KC139975 | gl0298, gl0299, gl0300, gl0301 |
| KC139826 | gl0373, gl0376 | KC139976 | gl0302 |
| KC139827 | Zxj-8 | KC139977 | gl0303, gl0304, gl0305, gl0307 |
| KC139828 | Lhm-M0505 | KC139978 | gl0306 |
| KC139829 | gl0288, gl0289, gl0292 | KC139979 | gl0308 |
| KC139830 | gl0290, gl0291 | KC139980 | gl0309 |
| KC139831 | gl0293, Zxj-9 | KC139981 | gl0310 |
| KC139832 | gl0294, gl0297 | KC139982 | gl0311 |
| KC139833 | gl0295 | KC139983 | gl0312 |
| KC139834 | gl0296 | KC139984 | Lbp-LC14 |
| KC139835 | sl0311 | KC139985 | gl0313 |
| KC139836 | sl0312 | KC139986 | gl0314, gl0315, gl0316, gl0317, gl0319 |
| KC139837 | sl0313 | KC139987 | gl0318 |
| KC139838 | sl0314 | KC139988 | gl0320, gl0321 |
| KC139839 | sl0315 | KC139989 | gl0322 |
| KC139840 | Lgd-02 | KC139990 | gl0323 |
| KC139841 | gl0272, gl0276 | KC139991 | gl0324 |
| KC139842 | gl0273 | KC139992 | gl0325 |
| KC139843 | gl0274 | KC139993 | gl0326 |
| KC139844 | gl0275 | KC139994 | gl0327, gl0328, gl0329, gl0330, gl0331 |
| KC139845 | Lbp-LC12 | KC139995 | sl0318, sl0319 |
| KC139846 | gl0277 | KC139996 | sl0320 |
| KC139847 | gl0278, gl0279, gl0281 | KC139997 | sl0321 |
| KC139848 | gl0280 | KC139998 | sl0322 |
| KC139849 | Lbp-LC13 | KC139999 | sl0323 |
| KC139850 | Cch-M0806 | KC140000 | sl0330 |
| KC139851 | gl0241 | KC140001 | sl0331 |
| KC139852 | gl0242 | KC140002 | sl0332 |
| KC139853 | gl0243 | KC140003 | sl0333 |
| KC139854 | gl0244 | KC140004 | sl0334 |
| KC139855 | gl0245 | KC140005 | Cch-M0807 |
| KC139856 | gl0246 | KC140006 | Cch-M0805 |
| KC139857 | gl0247 | KC140007 | Qzy-M0705 |
| KC139858 | gl0248, gl0249 | KC140008 | gl0221, gl0223 |
| KC139859 | gl0250 | KC140009 | gl0222 |
| KC139860 | gl0251 | KC140010 | gl0224 |
| KC139861 | gl0095, gl0227, gl0231, gl0233 | KC140011 | gl0225 |
| KC139862 | gl0096 | KC140012 | gl0226, Wj-HCO2 |
| KC139863 | gl0097, gl0238 | KC140013 | gl0332, gl0333, gl0335, gl0336 |
| KC139864 | gl0098 | KC140014 | gl0334 |
| KC139865 | gl0099, gl0232, gl0234 | KC140015 | Wsz-4 |
| KC139866 | gl0100 | KC140016 | Xll-2 |
| KC139867 | gl0228 | KC140017 | gl0168 |
| KC139868 | gl0229 | KC140018 | gl0169 |
| KC139869 | gl0230 | KC140019 | gl0170 |
| KC139870 | gl0235 | KC140020 | gl0171 |
| KC139871 | gl0236 | KC140021 | gl0172, gl0174, gl0175, gl0176, gl0177, gl0178, gl0179, gl0180, gl0181 gl0182, gl0183 |
| KC139872 | gl0237, gl0239 | KC140022 | gl0173 |
| KC139873 | gl0240 | KC140023 | gl0184 |
| KC139874 | gl0101 | KC140024 | gl0150 |
| KC139875 | gl0102, gl0105, gl0106, gl0115 | KC140025 | gl0151, gl0154, gl0155 |
| KC139876 | gl0103 | KC140026 | gl0152 |
| KC139877 | gl0104 | KC140027 | gl0153 |
| KC139878 | gl0107 | KC140028 | Zxj-12 |
| KC139879 | gl0108 | KC140029 | Wj-HC05 |
| KC139880 | gl0109 | KC140030 | gl0162 |
| KC139881 | gl0110 | KC140031 | gl0163, gl0187, Wj-HC04 |
| KC139882 | gl0111 | KC140032 | gl0164, gl0185, gl0191, gl0193, gl0195, gl0196, gl0197, gl0206, gl0215, gl0217, gl0220, Zxj-11a, Zxj-11b |
| KC139883 | gl0112 | KC140033 | gl0165 |
| KC139884 | gl0113 | KC140034 | gl0166 |
| KC139885 | gl0114 | KC140035 | gl0167, gl0216 |
| KC139886 | gl0089 | KC140036 | gl0186 |
| KC139887 | gl0090 | KC140037 | gl0188, gl0190, gl0156, gl0159 |
| KC139888 | gl0091 | KC140038 | gl0189 |
| KC139889 | gl0092 | KC140039 | gl0192, gl0199, gl0201, gl0203, gl0205, gl0207, gl0208, gl0210, gl0211, gl0212, gl0213, gl0214 |
| KC139890 | gl0093, gl0094 | KC140040 | gl0194 |
| KC139891 | gl0267 | KC140041 | gl0198 |
| KC139892 | gl0268 | KC140042 | gl0200 |
| KC139893 | gl0269 | KC140043 | gl0202 |
| KC139894 | gl0270 | KC140044 | gl0204 |
| KC139895 | gl0271 | KC140045 | gl0209 |
| KC139896 | gl0252, gl0254, gl0255 | KC140046 | gl0218 |
| KC139897 | gl0253 | KC140047 | gl0219 |
| KC139898 | gl0256, Zxj-4 | KC140048 | gl0157 |
| KC139899 | Zcy-2 | KC140049 | gl0158, gl0160, gl0161 |
| KC139900 | gl0083 | KC140050 | Wj-HC06 |
| KC139901 | gl0087 | KC140051 | gl0337, gl0338, gl0341 |
| KC139902 | gl0088 | KC140052 | gl0339, gl0340 |
| KC139903 | gl0077, gl0080 | KC140053 | gl0116, gl0118 |
| KC139904 | gl0078, gl0079, gl0081, gl0082, gl0084, gl0085, gl0086 | KC140054 | gl0117, gl0133, gl0135, gl0136, gl0137 |
| KC139905 | gl0071 | KC140055 | gl0119 |
| KC139906 | gl0072 | KC140056 | gl0120 |
| KC139907 | gl0073 | KC140057 | gl0121, gl0124, gl0128, Wj-HC03 |
| KC139908 | gl0074 | KC140058 | gl0122 |
| KC139909 | gl0075 | KC140059 | gl0123 |
| KC139910 | gl0076 | KC140060 | gl0125 |
| KC139911 | gl0059, gl0063 | KC140061 | gl0126 |
| KC139912 | gl0060, gl0064 | KC140062 | gl0127, gl0129 |
| KC139913 | gl0061 | KC140063 | gl0130 |
| KC139914 | gl0062 | KC140064 | gl0131 |
| KC139915 | gl0065 | KC140065 | gl0132 |
| KC139916 | gl0066 | KC140066 | gl0134 |
| KC139917 | gl0067 | KC140067 | gl0138, gl0139 |
| KC139918 | gl0068 | KC140068 | gl0140 |
| KC139919 | gl0069 | KC140069 | gl0141 |
| KC139920 | gl0070 | KC140070 | gl0142 |
| KC139921 | gl0053 | KC140071 | gl0143 |
| KC139922 | gl0054 | KC140072 | gl0144 |
| KC139923 | gl0055 | KC140073 | gl0145 |
| KC139924 | gl0056 | KC140074 | gl0146 |
| KC139925 | gl0057 | KC140075 | gl0147 |
| KC139926 | gl0058 | KC140076 | gl0149 |
| KC139927 | gl0047, gl0051 | KC140077 | sl0326 |
| KC139928 | gl0048 | KC140078 | sl0327 |
| KC139929 | gl0049 | KC140079 | sl0328 |
| KC139930 | gl0050 | KC140080 | sl0329 |
| KC139931 | gl0052 | KC140081 | Gj-M0802 |
| KC139932 | gl0017, gl0019, gl0020 | KC140082 | sl0335 |
| KC139933 | gl0021, gl0022 | KC140083 | sl0336, sl0339 |
| KC139934 | gl0026 | KC140084 | sl0337 |
| KC139935 | gl0023 | KC140085 | sl0338 |
| KC139936 | gl0024 | KC140086 | sl0340 |
| KC139937 | gl0025 | KC140087 | sl0341 |
| KC139938 | gl0027 | KC140088 | sl0342 |
| KC139939 | gl0001, gl0016 | KC140089 | sl0343 |
| KC139940 | gl0002, gl0014, gl0015 | KC140090 | Ln-M0501 |
| KC139941 | gl0003, gl0012 | KC140091 | Xll-M0631 |
| KC139942 | gl0004, gl0011 | KC140092 | Zxj-17 |
| KC139943 | gl0005 | KC140093 | Gj-M0803 |
| KC139944 | gl0006 | KC140094 | Zxj-30 |
| KC139945 | gl0007, gl0009 | KC140095 | Ly-2 |
| KC139946 | gl0008 | KC140096 | Zxj-29 |
| KC139947 | gl0010 | KC140097 | Hj-M0502 |
| KC139948 | gl0013 | KC140098 | Gj-M0801 |
| KC139949 | gl0018 | KC140099 | Xll-M0632 |
| KC139950 | gl0029 | KC140100 | Zxj-39 |
| KC139951 | gl0030, gl0034 | KC140101 | Wsz-1 |
| KC139952 | gl0031, gl0033 |  |  |
