# Supplementary material for: DNA Barcoding and Species Boundary Delimitation of Selected Species of Chinese Acridoidea (Orthoptera: Caelifera)
Source: PLoS One. 2013 Dec 20;8(12):e82400. doi: 10.1371/journal.pone.0082400 (PMC3869712; doi:10.1371/journal.pone.0082400)
Supplement: Table S4 — ranges of intraspecific genetic variations. (DOC) [file pone.0082400.s007.doc]

**Table S4. Ranges of intraspecific genetic variations**

| Species (number of  populations sampled) | Intraspecific variations | Species (number of populations sampled) | Intraspecific variations |
| --- | --- | --- | --- |
| *Calliptamus abbreviatus* (13) | 0~1.08% | *Prumna arcticus* (2) | 0.3~2.17% |
| *Calliptamus barbarus* (1) | 0~0.46% | *Tonkinacris sinensis* (1) | 0~0.3% |
| *Calliptamus italicus* (2) | 0~0.3% | *Diobolocatantops pingius* (2) | 0~0.3% |
| *Traulia minuta* (1) | 0~2.01% | *Stenocatantops splendens* (1) | 0~0.3% |
| *Shirakiacris shirakii* (2) | 0~1.7% | *Xenocatantops brachycerus* (2) | 0.15~1.07% |
| *Shirakiacris yunkweiensis* (2) | 0~1.54% | *Spathosternum prasiniferum sinense* (1) | 0~0.3% |
| ***Shirakiacris shirakii+yunkweiensis* (4) ***** | 0~2.01% | *Spathosternum prasiniferum* *prasiniferum* (1) | 0.15~0.61% |
| *Emeiacris maculatus* (1) | 0~0. 61% | *Toacris yashanensis* (1) | 0 |
| *Paratonkinacris vittifemoralis* (1) | 0~1.38% | *Pseudoxya diminuta* (2) | 0~1.07% |
| *Fruhstorferiola huayinensis* (4) | 0~1.85% | *Oxya sp* (1) | 0.61~2.01% |
| *Fruhstorferiola kulinga* (4) | 0~2.97% | *Oxya chinensis* (2) | 0.15~0.46% |
| ***Fruhstorferiola huayinensis+kulinga* (8) ***** | 0~2.97% | *Aiolopus tamulus* (2) | 0~2.01% |
| *Fruhstorferiola tonkinensis* (1) | 0~0.77% | *Locusta migratoria* (1) | 0~2.96% |
| *Indopodisma kindoni* (1) | 0.46~1.23% | *Oedaleus decorus* (3) | 0~2.17% |
| *Ognevia longipennis* (3) | 0~0.46% | *Oedaleus asiaticus* (6) | 0~1.23% |
| *Pedopodisma funiushana* (1) | 0~0.76% | ***Oedaleus decorus +asiaticus* (9) ***** | 0~2.17% |
| *Pedopodisma tsinlingensis* (1) | 0~0.15% | *Oedaleus infernalis* (11) | 0~2.47% |
| *Pedopodisma wudangshanensis* (1) | 0.15~2.17% | *Oedaleus manjius* (2) | 0~1.7% |
| ***Pedopodisma funiushana+tsinlingensis***  ***+ wudangshanensis* (3) ***** | 0~2.33% | ***Oedaleus infernalis +manjius* (13) ***** | 0~2.63% |
| *Sinopodisma houshana* (2) | 0~2.17% | *Oedaleus abrauptus* (1) | 0~0.15% |
| *Sinopodisma lushiensis* (1) | 0.15~0.61% | *Trilophidia annulata* (11) | 0~2.33% |
| *Sinopodisma qinlingensis* (1) | 0~0.61% | *Pternoscirta caliginosa* (1) | 0.15~1.07% |
| ***Sinopodisma houshana+lushiensis+qinlingensis* (4) ***** | 0~2.17% | *Omocestus haemorrhoidalis* (1) | 0~0.92% |
| *Sinopodisma lofaoshana* (2) | 0~0.92% (within population)  5.06~5.56% (between population) | *Euchorthippus unicolor* (1) | 0~0.61% |
| *Sinopodisma rostellocerca* (4) | 0~2.49% | *Chorthippus parallelus* (1) | 1.85% |
| *Sinopodisma wulingshana* (3) | 0~2.32% | *Mongolotettix japonicus* (2) | 1.54% |

* The genetic distances between individuals of the same population are always less than 1%, and those between conspecific individuals from different populations of the same species usually less than 3%. The asterisk “*” indicates the species group which might be considered as the same species according to the morphological similarities.
