# Supplementary material for: DNA Barcoding and Species Boundary Delimitation of Selected Species of Chinese Acridoidea (Orthoptera: Caelifera)
Source: PLoS One. 2013 Dec 20;8(12):e82400. doi: 10.1371/journal.pone.0082400 (PMC3869712; doi:10.1371/journal.pone.0082400)
Supplement: Table S5 — Distribution of intraspecific variations and interspecific divergences. (DOC) [file pone.0082400.s008.doc]

**Table S5. Distribution of intraspecific variations and interspecific divergences**

| Distribution of intraspecific variation | | | | Distribution of interspecific divergence within a genus | | | |
| --- | --- | --- | --- | --- | --- | --- | --- |
| Distance range | frequency | percentage | cumulative | Distance range | frequency | percentage | cumulative |
| <= 0.0% | 422 | 10.75% | 10.75% | <= 0.0% | 16 | 0.29% | 0.29% |
| 0.0% to 0.5% | 1879 | 47.83% | 58.58% | 0.0% to 1.0% | 555 | 10.26% | 10.55% |
| 0.5% to 1.0% | 854 | 21.76% | 80.34% | 1.0% to 2.0% | 475 | 8.77% | 19.32% |
| 1.0% to 1.5% | 338 | 8.6% | 88.94% | 2.0% to 3.0% | 172 | 3.18% | 22.5% |
| 1.5% to 2.0% | 216 | 5.49% | 94.43% | 3.0% to 4.0% | 121 | 2.24% | 24.74% |
| 2.0% to 2.5% | 191 | 4.86% | 99.29% | 4.0% to 5.0% | 382 | 7.05% | 31.79% |
| 2.5% to 3.0% | 4 | 0.1% | 99.39% | 5.0% to 6.0% | 1286 | 23.77% | 55.56% |
| 5.0% to 5.5% | 20 | 0.51% | 99.9% | 6.0% to 7.0% | 393 | 7.26% | 62.82% |
| 5.5% to 6.0% | 4 | 0.1% | 100% | 7.0% to 8.0% | 261 | 4.82% | 67.64% |
|  |  |  |  | 8.0% to 9.0% | 1428 | 26.39% | 94.03% |
|  |  |  |  | 9.0% to 10.0% | 191 | 3.54% | 97.56% |
|  |  |  |  | 10.0% to 11.0% | 126 | 2.32% | 99.88% |
|  |  |  |  | 11.0% to 12.0% | 4 | 0.08% | 99.96% |
|  |  |  |  | 12.0% to 13.0% | 1 | 0.02% | 99.98% |
|  |  |  |  | 13.0% to 14.0% | 1 | 0.02% | 100% |
