# Supplementary material for: DNA Barcoding and Species Boundary Delimitation of Selected Species of Chinese Acridoidea (Orthoptera: Caelifera)
Source: PLoS One. 2013 Dec 20;8(12):e82400. doi: 10.1371/journal.pone.0082400 (PMC3869712; doi:10.1371/journal.pone.0082400)
Supplement: Table S6 — Sequences identified as ambiguous or incorrect in BCM analysis. (DOC) [file pone.0082400.s009.doc]

**Table S6 Sequences identified as ambiguous or incorrect in BCM analysis**

| **Code of query sequence** | **Morphospecies name** | **Identification** |
| --- | --- | --- |
| gl0240 | *Fruhstorferiola huayinensis* | Incorrect |
| gl0107 | *Fruhstorferiola kulinga* | Incorrect |
| gl0108 | *Fruhstorferiola kulinga* | Incorrect |
| gl0109 | *Fruhstorferiola kulinga* | Incorrect |
| gl0153 | *Oedaleus decorus* | Incorrect |
| Wj-HC05 | *Oedaleus decorus* | Incorrect |
| Zxj-12 | *Oedaleus decorus* | Incorrect |
| gl0157 | *Oedaleus manjius* | Incorrect |
| Wj-HC06 | *Oedaleus manjius* | Incorrect |
| FJ531686 | *Pedopodisma funiushana* | Incorrect |
| gl0273 | *Shirakiacris shirakii* | Incorrect |
| gl0049 | *Sinopodisma qinlingensis* | Incorrect |
| gl0100 | *Fruhstorferiola huayinensis* | Ambiguous |
| gl0188 | *Oedaleus infernalis* | Ambiguous |
| gl0190 | *Oedaleus infernalis* | Ambiguous |
| gl0198 | *Oedaleus infernalis* | Ambiguous |
| gl0202 | *Oedaleus infernalis* | Ambiguous |
| gl0209 | *Oedaleus infernalis* | Ambiguous |
| Wj-HC04 | *Oedaleus infernalis* | Ambiguous |
| gl0156 | *Oedaleus manjius* | Ambiguous |
| gl0159 | *Oedaleus manjius* | Ambiguous |
| gl0083 | *Pedopodisma funiushana* | Ambiguous |
| gl0084 | *Pedopodisma funiushana* | Ambiguous |
| gl0085 | *Pedopodisma funiushana* | Ambiguous |
| gl0086 | *Pedopodisma funiushana* | Ambiguous |
| gl0087 | *Pedopodisma funiushana* | Ambiguous |
| gl0088 | *Pedopodisma funiushana* | Ambiguous |
| gl0078 | *Pedopodisma tsinlingensis* | Ambiguous |
| gl0079 | *Pedopodisma tsinlingensis* | Ambiguous |
| gl0081 | *Pedopodisma tsinlingensis* | Ambiguous |
| gl0082 | *Pedopodisma tsinlingensis* | Ambiguous |
| gl0280 | *Shirakiacris yunkweiensis* | Ambiguous |
| gl0053 | *Sinopodisma lushiensis* | Ambiguous |
| gl0058 | *Sinopodisma lushiensis* | Ambiguous |
| gl0048 | *Sinopodisma qinlingensis* | Ambiguous |
