# Supplementary material for: DNA Barcoding and Species Boundary Delimitation of Selected Species of Chinese Acridoidea (Orthoptera: Caelifera)
Source: PLoS One. 2013 Dec 20;8(12):e82400. doi: 10.1371/journal.pone.0082400 (PMC3869712; doi:10.1371/journal.pone.0082400)
Supplement: Table S7 — Assignment of species through BP-based method. (DOC) [file pone.0082400.s010.doc]

**Table S7. Assignment of species through BP-based method.**

| Query sequence code | Predicted species | Probability |
| --- | --- | --- |
| #1 C_abb285 | *Calliptamus abbreviatus* | 0.942349 |
| #2 C_abb286 | *Calliptamus abbreviatus* | 0.984127 |
| #3 C_abb287 | *Calliptamus abbreviatus* | 0.984675 |
| #4 C_abb345 | *Calliptamus abbreviatus* | 0.978831 |
| #5 C_abb346 | *Calliptamus abbreviatus* | 0.989758 |
| #6 C_abb349 | *Calliptamus abbreviatus* | 0.988526 |
| #7 C_abb350 | *Calliptamus abbreviatus* | 0.990107 |
| #8 C_abb351 | *Calliptamus abbreviatus* | 0.98952 |
| #9 C_abb353 | *Calliptamus abbreviatus* | 0.988123 |
| #10 C_abb356 | *Calliptamus abbreviatus* | 0.990981 |
| #11 C_abb359 | *Calliptamus abbreviatus* | 0.988024 |
| #12 C_abb360 | *Calliptamus abbreviatus* | 0.988123 |
| #13 C_abb361 | *Calliptamus abbreviatus* | 0.988123 |
| #14 C_abb365 | *Calliptamus abbreviatus* | 0.988123 |
| #15 C_abb366 | *Calliptamus abbreviatus* | 0.988123 |
| #16 C_abb369 | *Calliptamus abbreviatus* | 0.989089 |
| #17 C_abb370 | *Calliptamus abbreviatus* | 0.988123 |
| #18 C_abb371 | *Calliptamus abbreviatus* | 0.988459 |
| #19 C_abb375 | *Calliptamus abbreviatus* | 0.988123 |
| #20 C_abb376 | *Calliptamus abbreviatus* | 0.986713 |
| #21 C_abb_Lhm | *Calliptamus abbreviatus* | 0.986894 |
| #22 C_abb_zxj | *Calliptamus abbreviatus* | 0.989757 |
| #23 C_bar291 | *Calliptamus barbarus* | 0.959281 |
| #24 C_bar292 | *Calliptamus abbreviatus* | 0.965733 |
| #25 C_ita297 | *Calliptamus italicus* | 0.975577 |
| #26 C_ita_NC_011305 | *Calliptamus italicus* | 0.975563 |
| #27 C_ita_zxj | *Calliptamus italicus* | 0.980694 |
| #28 E_mac244 | *Emeiacris maculata* | 0.974642 |
| #29 E_mac245 | *Emeiacris maculata* | 0.974519 |
| #30 E_mac246 | *Emeiacris maculata* | 0.97235 |
| #31 P_vit250 | *Paratonkinacris vittifemoralis* | 0.986012 |
| #32 P_vit251 | *Paratonkinacris vittifemoralis* | 0.983906 |
| #33 F_hua098 | *Fruhstorferiola huayinensis* | 0.511742 |
| #34 F_hua099 | *Fruhstorferiola huayinensis* | 0.975892 |
| #35 F_hua100 | *Fruhstorferiola huayinensis* | 0.49754 |
| #36 F_hua230 | *Fruhstorferiola huayinensis* | 0.989397 |
| #37 F_hua231 | *Fruhstorferiola huayinensis* | 0.988623 |
| #38 F_hua233 | *Fruhstorferiola huayinensis* | 0.988623 |
| #39 F_hua234 | *Fruhstorferiola huayinensis* | 0.975892 |
| #40 F_hua238 | *Fruhstorferiola huayinensis* | 0.984397 |
| #41 F_hua239 | *Fruhstorferiola huayinensis* | 0.981827 |
| #42 F_hua240 | *Fruhstorferiola kulinga* | 0.985128 |
| #43 F_kul104 | *Fruhstorferiola kulinga* | 0.988717 |
| #44 F_kul105 | *Fruhstorferiola kulinga* | 0.988874 |
| #45 F_kul106 | *Fruhstorferiola kulinga* | 0.988874 |
| #46 F_kul108 | *Fruhstorferiola huayinensis* | 0.953346 |
| #47 F_kul111 | *Fruhstorferiola kulinga* | 0.967439 |
| #48 F_kul112 | *Fruhstorferiola kulinga* | 0.967786 |
| #49 F_kul115 | *Fruhstorferiola kulinga* | 0.988874 |
| #50 F_ton092 | *Fruhstorferiola tonkinensis* | 0.967645 |
| #51 F_ton093 | *Fruhstorferiola tonkinensis* | 0.976889 |
| #52 F_ton094 | *Fruhstorferiola tonkinensis* | 0.976889 |
| #53 I_kin270 | *Indopodisma kingdoni* | 0.983393 |
| #54 I_kin271 | *Indopodisma kingdoni* | 0.975119 |
| #55 O_lon256 | *Ognevia longipennis* | 0.973263 |
| #56 O_lon_zxj | *Ognevia longipennis* | 0.973263 |
| #57 O_lon_zcy | *Ognevia longipennis* | 0.961679 |
| #58 P_fun_084 | *Pedopodisma tsinlingensis* | 0.973709 |
| #59 P_fun_085 | *Pedopodisma tsinlingensis* | 0.973709 |
| #60 P_fun_086 | *Pedopodisma tsinlingensis* | 0.973709 |
| #61 P_tsi_080 | *Pedopodisma tsinlingensis* | 0.972226 |
| #62 P_tsi_081 | *Pedopodisma tsinlingensis* | 0.973709 |
| #63 P_tsi_082 | *Pedopodisma tsinlingensis* | 0.973709 |
| #64 P_wud_074 | *Pedopodisma funiusha* | 0.968561 |
| #65 P_wud_075 | *Pedopodisma wudangshanensis* | 0.942986 |
| #66 P_wud_076 | *Pedopodisma funiusha* | 0.892706 |
| #67 S_hou_062 | *Sinopodisma houshana* | 0.952429 |
| #68 S_hou_063 | *Sinopodisma houshana* | 0.971164 |
| #69 S_hou_064 | *Sinopodisma houshana* | 0.976509 |
| #70 S_hou_068 | *Sinopodisma houshana* | 0.978234 |
| #71 S_hou_069 | *Sinopodisma houshana* | 0.979464 |
| #72 S_hou_070 | *Sinopodisma houshana* | 0.975178 |
| #73 S_lus_056 | *Sinopodisma lushiensis* | 0.957328 |
| #74 S_lus_057 | *Sinopodisma lushiensis* | 0.952633 |
| #75 S_lus_058 | *Sinopodisma lushiensis* | 0.536554 |
| #76 S_qin_050 | *Sinopodisma qinlingensis* | 0.952128 |
| #77 S_qin_051 | *Sinopodisma qinlingensis* | 0.979646 |
| #78 S_qin_052 | *Sinopodisma qinlingensis* | 0.943538 |
| #79 S_lof021 | *Sinopodisma lofaoshana* | 0.973749 |
| #80 S_lof022 | *Sinopodisma lofaoshana* | 0.973749 |
| #81 S_lof026 | *Sinopodisma lofaoshana* | 0.957353 |
| #82 S_lof025 | *Sinopodisma lofaoshana* | 0.973094 |
| #83 S_lof027 | *Sinopodisma lofaoshana* | 0.973583 |
| #84 S_ros_006 | *Sinopodisma rostellocerca* | 0.973192 |
| #85 S_ros_009 | *Sinopodisma rostellocerca* | 0.990078 |
| #86 S_ros_010 | *Sinopodisma rostellocerca* | 0.983077 |
| #87 S_ros_012 | *Sinopodisma rostellocerca* | 0.98374 |
| #88 S_ros_013 | *Sinopodisma rostellocerca* | 0.982853 |
| #89 S_ros_015 | *Sinopodisma rostellocerca* | 0.988624 |
| #90 S_ros_016 | *Sinopodisma rostellocerca* | 0.989035 |
| #91 S_wul_032 | *Sinopodisma wulingshana* | 0.933971 |
| #92 S_wul_033 | *Sinopodisma wulingshana* | 0.981355 |
| #93 S_wul_034 | *Sinopodisma wulingshana* | 0.98537 |
| #94 S_wul_038 | *Sinopodisma wulingshana* | 0.977013 |
| #95 S_wul_039 | *Sinopodisma wulingshana* | 0.943744 |
| #96 S_wul_040 | *Sinopodisma wulingshana* | 0.955066 |
| #97 S_wul_044 | *Sinopodisma wulingshana* | 0.984185 |
| #98 S_wul_045 | *Sinopodisma wulingshana* | 0.955145 |
| #99 S_wul_046 | *Sinopodisma wulingshana* | 0.969427 |
| #100 P_arc265 | *Prumna arctica* | 0.972714 |
| #101 P_arc266 | *Prumna arctica* | 0.95596 |
| #102 P_arc_FJ531674 | *Prumna arctica* | 0.958999 |
| #103 T_sin260 | *Tonkinacris sinensis* | 0.960067 |
| #104 T_sin261 | *Tonkinacris sinensis* | 0.965169 |
| #105 T_min_sl0315 | *Traulia minuta* | 0.970592 |
| #106 T_min_Lgd | *Traulia minuta* | 0.975628 |
| #107 T_min_FJ571149 | *Traulia minuta* | 0.968252 |
| #108 S_shi275 | *Shirakiacris shirakii* | 0.956174 |
| #109 S_shi276 | *Shirakiacris shirakii* | 0.960738 |
| #110 S_shi_Lbp | *Shirakiacris shirakii* | 0.957122 |
| #111 S_yun280 | *Shirakiacris shirakii* | 0.543701 |
| #112 S_yun281 | *Shirakiacris yunkweiensis* | 0.968045 |
| #113 S_yun_Lbp | *Shirakiacris shirakii* | 0.912641 |
| #114 D_pin301 | *Diabolocatantops pinguis* | 0.967153 |
| #115 D_pin302 | *Diabolocatantops pinguis* | 0.972652 |
| #116 S_spl306 | *Stenocatantops splendens* | 0.947883 |
| #117 S_spl307 | *Stenocatantops splendens* | 0.965141 |
| #118X_bra311 | *Xenocatantops brachycerus* | 0.977701 |
| #119X_bra312 | *Xenocatantops brachycerus* | 0.976412 |
| #120X_bra_Lbp | *Xenocatantops brachycerus* | 0.976921 |
| #121 S_sin316 | *Spathosternum prasiniferum sinense* | 0.974541 |
| #122 S_sin317 | *Spathosternum prasiniferum sinense* | 0.974541 |
| #123 S_sin320 | *Spathosternum prasiniferum sinense* | 0.972301 |
| #124 S_sin321 | *Spathosternum prasiniferum sinense* | 0.972301 |
| #125 S_pra325 | *Spathosternum prasiniferum prasiniferum* | 0.966416 |
| #126 S_pra326 | *Spathosternum prasiniferum prasiniferum* | 0.972269 |
| #127 T_yao330 | *Toacris yaoshanensis* | 0.963296 |
| #128 T_yao331 | *Toacris yaoshanensis* | 0.963296 |
| #129 P_dim_sl0320 | *Pseudoxya diminuta* | 0.988669 |
| #130 P_dim_sl0321 | *Pseudoxya diminuta* | 0.987679 |
| #131 P_dim_sl0324 | *Pseudoxya diminuta* | 0.987821 |
| #132 P_dim_sl0325 | *Pseudoxya diminuta* | 0.987821 |
| #133 O_chi_NC_010219 | *Oxya chinensis* | 0.970709 |
| #134 A_tam224 | *Aiolopus tamulus* | 0.97805 |
| #1351 A_tam225 | *Aiolopus tamulus* | 0.982141 |
| #136 A_tam226 | *Aiolopus tamulus* | 0.970058 |
| #137 A_tam335 | *Aiolopus tamulus* | 0.973667 |
| #138 A_tam336 | *Aiolopus tamulus* | 0.973667 |
| #139 A_tam_Wj | *Aiolopus tamulus* | 0.970058 |
| #140 L_mig_Xll | *Locusta migratoria* | 0.955747 |
| #141 O_asi171 | *Oedaleus asiaticus* | 0.968128 |
| #142 O_asi172 | *Oedaleus asiaticus* | 0.98822 |
| #143 O_asi173 | *Oedaleus asiaticus* | 0.95671 |
| #144 O_asi177 | *Oedaleus asiaticus* | 0.98822 |
| #145 O_asi178 | *Oedaleus asiaticus* | 0.98822 |
| #146 O_asi179 | *Oedaleus asiaticus* | 0.98822 |
| #147 O_asi182 | *Oedaleus asiaticus* | 0.98822 |
| #148 O_asi183 | *Oedaleus asiaticus* | 0.98822 |
| #149 O_dec153 | *Oedaleus asiaticus* | 0.836449 |
| #150 O_dec154 | *Oedaleus decorus* | 0.95852 |
| #151 O_dec155 | *Oedaleus decorus* | 0.95852 |
| #152 O_dec_Wj | *Oedaleus asiaticus* | 0.810996 |
| #153 O_inf165 | *Oedaleus infernalis* | 0.975717 |
| #154 O_inf166 | *Oedaleus infernalis* | 0.97515 |
| #155 O_inf167 | *Oedaleus infernalis* | 0.978689 |
| #156 O_inf186 | *Oedaleus infernalis* | 0.913264 |
| #157 O_inf189 | *Oedaleus infernalis* | 0.980189 |
| #158 O_inf190 | *Oedaleus infernalis* | 0.980211 |
| #159 O_inf194 | *Oedaleus infernalis* | 0.97929 |
| #160 O_inf195 | *Oedaleus infernalis* | 0.979851 |
| #161 O_inf196 | *Oedaleus infernalis* | 0.979851 |
| #162 O_inf200 | *Oedaleus infernalis* | 0.871336 |
| #163 O_inf201 | *Oedaleus infernalis* | 0.983077 |
| #164 O_inf202 | *Oedaleus infernalis* | 0.967786 |
| #165 O_inf206 | *Oedaleus infernalis* | 0.979851 |
| #166 O_inf207 | *Oedaleus infernalis* | 0.983077 |
| #167 O_inf208 | *Oedaleus infernalis* | 0.983077 |
| #168 O_inf212 | *Oedaleus infernalis* | 0.983077 |
| #169 O_inf213 | *Oedaleus infernalis* | 0.983077 |
| #170 O_inf214 | *Oedaleus infernalis* | 0.983077 |
| #171 O_inf218 | *Oedaleus infernalis* | 0.97312 |
| #172 O_inf219 | *Oedaleus infernalis* | 0.967184 |
| #173 O_inf220 | *Oedaleus infernalis* | 0.979851 |
| #174 O_inf_Wj | *Oedaleus infernalis* | 0.949014 |
| #175 O_man156 | *Oedaleus infernalis* | 0.980211 |
| #176 O_man159 | *Oedaleus infernalis* | 0.980211 |
| #177 O_man161 | *Oedaleus manjius* | 0.974857 |
| #178 O_abr340 | *Oedaleus abruptus* | 0.970211 |
| #179 O_abr341 | *Oedaleus abruptus* | 0.97189 |
| #180 T_ann119 | *Trilophidia annulata* | 0.992431 |
| #181 T_ann120 | *Trilophidia annulata* | 0.99051 |
| #182 T_ann121 | *Trilophidia annulata* | 0.992201 |
| #183 T_ann125 | *Trilophidia annulata* | 0.993663 |
| #184 T_ann126 | *Trilophidia annulata* | 0.988524 |
| #185 T_ann128 | *Trilophidia annulata* | 0.992201 |
| #186 T_ann129 | *Trilophidia annulata* | 0.992064 |
| #187 T_ann131 | *Trilophidia annulata* | 0.991696 |
| #188 T_ann135 | *Trilophidia annulata* | 0.991248 |
| #189 T_ann136 | *Trilophidia annulata* | 0.991248 |
| #190 T_ann137 | *Trilophidia annulata* | 0.991248 |
| #191 T_ann141 | *Trilophidia annulata* | 0.992877 |
| #192 T_ann142 | *Trilophidia annulata* | 0.991527 |
| #193 T_ann143 | *Trilophidia annulata* | 0.992305 |
| #194 T_ann145 | *Trilophidia annulata* | 0.992925 |
| #195 T_ann148 | *Trilophidia annulata* | 0.991248 |
| #196 T_ann149 | *Trilophidia annulata* | 0.991992 |
| #197 P_cal_sl0328 | *Pternoscirta caliginosa* | 0.976905 |
| #198 P_cal_sl0329 | *Pternoscirta caliginosa* | 0.976517 |
| #199 O_hae_sl0338 | *Omocestus haemorrhoidalis* | 0.971946 |
| #200 O_hae_sl0339 | *Omocestus haemorrhoidalis* | 0.974353 |
| #201 E_uni_sl0343 | *Euchorthippus unicolor* | 0.980456 |
| #202 E_uni_sl0344 | *Euchorthippus unicolor* | 0.980456 |
